# Treatment With Icosapent Ethyl to Reduce Ischemic Events in Patients With Prior Percutaneous Coronary Intervention: Insights From REDUCE‐IT PCI

**DOI:** 10.1161/JAHA.121.022937

**Published:** 2022-03-09

**Authors:** Benjamin E. Peterson, Deepak L. Bhatt, Ph. Gabriel Steg, Michael Miller, Eliot A. Brinton, Terry A. Jacobson, Steven B. Ketchum, Rebecca A. Juliano, Lixia Jiao, Ralph T. Doyle, Craig Granowitz, C. Michael Gibson, Duane Pinto, Robert P. Giugliano, Matthew J. Budoff, Jean‐Claude Tardif, Subodh Verma, Christie M. Ballantyne, the REDUCE‐IT Investigators

**Affiliations:** ^1^ Brigham and Women’s Hospital Heart and Vascular Center Harvard Medical School Boston MA; ^2^ Université de Paris AP‐HP (Assistance Publique‐Hôpitaux de Paris) Hôpital Bichat FACT (French Alliance for Cardiovascular Trials) INSERM U‐1148 Paris France; ^3^ Department of Medicine University of Maryland School of Medicine Baltimore MD; ^4^ Utah Lipid Center Salt Lake City UT; ^5^ Department of Medicine Office of Health Promotion and Disease Prevention Emory University School of Medicine Atlanta GA; ^6^ Amarin Pharma, Inc. (Amarin) Bridgewater NJ; ^7^ Baim Clinical Research Institute Boston MA; ^8^ David Geffen School of Medicine Lundquist Institute Torrance CA; ^9^ Montreal Heart Institute Université de Montréal Quebec Canada; ^10^ Division of Cardiac Surgery St Michael’s Hospital University of Toronto Ontario Canada; ^11^ Department of Medicine Baylor College of Medicine Center for Cardiovascular Disease Prevention Methodist DeBakey Heart and Vascular Center Houston TX

**Keywords:** eicosapentaenoic acid, icosapent ethyl, prevention, revascularization, Percutaneous Coronary Intervention, Stent, Treatment

## Abstract

**Background:**

Patients who undergo percutaneous coronary intervention (PCI) are at increased risk for recurrent cardiovascular events despite aggressive medical therapy.

**Methods and Results:**

This post hoc analysis focused on the subset of patients with prior PCI enrolled in REDUCE‐IT (Reduction of Cardiovascular Events With Icosapent Ethyl–Intervention Trial), a multicenter, randomized, double‐blind, placebo‐controlled trial of icosapent ethyl versus placebo. Icosapent ethyl was added to statins in patients with low‐density lipoprotein cholesterol <100 mg/dL and fasting triglycerides 135–499 mg/dL. The primary end point was a composite of cardiovascular death, nonfatal myocardial infarction, nonfatal stroke, coronary revascularization, or unstable angina requiring hospitalization. There were 8179 patients randomized in REDUCE‐IT followed for a median of 4.9 years, and 3408 (41.7%) of them had a prior PCI with a median follow‐up of 4.8 years. These patients were randomized a median of 2.9 years (11 days to 30.7 years) after PCI. Among patients treated with icosapent ethyl versus placebo, there was a 34% reduction in the primary composite end point (hazard ratio [HR], 0.66; 95% CI, 0.58–0.76; *P*<0.001; number needed to treat^4.8 years^=12) and a 34% reduction in the key secondary composite end point of cardiovascular death, nonfatal myocardial infarction, or nonfatal stroke (HR, 0.66; 95% CI, 0.56–0.79; *P*<0.001; NNT^4.8 years^=19) versus placebo. Similarly, large reductions occurred in total coronary revascularizations and revascularization subtypes. There was also a 39% reduction in total events (rate ratio, 0.61; 95% CI, 0.52–0.72; *P*<0.001).

**Conclusions:**

Among patients treated with statins with elevated triglycerides and a history of prior PCI, icosapent ethyl substantially reduced the risk of recurrent events during an average of ~5 years of follow‐up with a number needed to treat of only 12.

**Registration:**

URL: https://www.clinicaltrials.gov; Unique identifier: NCT01492361.

**Nonstandard Abbreviations and Acronyms**
EPA: eicosapentaenoic acid
MACE: major adverse cardiovascular events
NNT: number needed to treat
REDUCE‐IT: Reduction of Cardiovascular Events With Icosapent Ethyl–Intervention Trial
STRENGTH: Long‐Term Outcomes Study to Assess Statin Residual Risk With Epanova in High Cardiovascular Risk Patients With Hypertriglyceridemia

**Clinical Perspective**

**What Is New?**

- Icosapent ethyl greatly reduced first occurrences of cardiovascular events among patients who had elevated triglycerides despite statin therapy and a history of prior percutaneous coronary intervention; number needed to treat^4.8 years^=12.
- There were also significant reductions in total ischemic events (first and subsequent), cardiovascular death, nonfatal myocardial infarction, nonfatal stroke, hospitalization for unstable angina, and coronary revascularization.

**What Are the Clinical Implications?**

- Patients with prior percutaneous coronary intervention and elevated triglycerides despite statin therapy are at extremely high risk for recurrent cardiovascular events.
- Icosapent ethyl could benefit a large proportion of patients with a history of prior percutaneous coronary intervention, and such patients should be screened for eligibility.

Patients who undergo percutaneous coronary intervention (PCI) are at increased risk for subsequent cardiovascular events when compared with patients with other cardiovascular risk factors.^1^ In recent years, efforts to improve stent design, lower low‐density lipoprotein cholesterol, and modify inflammation and platelet activity have resulted in some reductions in repeat events among patients who undergo coronary stenting.^2^, ^3^, ^4^ Yet, many patients still experience recurrent events, especially those with diabetes and elevated triglycerides.^5^, ^6^, ^7^, ^8^

The REDUCE‐IT (Reduction of Cardiovascular Events With Icosapent Ethyl–Intervention Trial) trial was designed to test the effectiveness of icosapent ethyl 4 g/day (a highly purified form of eicosapentaenoic acid [EPA]) versus placebo among patients with established cardiovascular disease or diabetes and additional risk factors.^9^, ^10^ The significant reduction in first and total major adverse cardiovascular events (MACE) among patients who were treated with icosapent ethyl was out of proportion to the degree of reduction in triglycerides.^11^, ^12^, ^13^, ^14^ These large reductions occurred in patients with diabetes, patients with only modestly elevated triglycerides, patients in the United States, and patients across numerous other prespecified subgroups.^15^, ^16^, ^17^, ^18^ Treatment with icosapent ethyl also substantially reduced instances of first and subsequent revascularization events.^19^, ^20^, ^21^

The aim of the present post hoc analysis of the REDUCE‐IT trial was to study the effects of icosapent ethyl versus placebo among patients who have been treated previously with PCI.

## Methods

The data that support the findings of this study may be made available from the corresponding author on reasonable request.

### Patient Population and Treatment

The design of the REDUCE‐IT trial has been published previously.^9^ REDUCE‐IT was a double‐blind, multicenter, placebo‐controlled, randomized trial comparing the effects of icosapent ethyl in high‐risk patients treated with statins with persistently elevated triglycerides. After a screening period of up to 60 days, patients were randomized to receive icosapent ethyl 4 g daily (2 g twice daily) versus a matching placebo.

Patients were enrolled in REDUCE‐IT if they were at least 45 years of age and had established cardiovascular disease or at least 50 years of age and had diabetes and additional risk factors. In this present post hoc analysis, patients were analyzed only if they had a prior PCI, such as balloon angioplasty or stenting (drug‐eluting or bare‐metal stents). Patients were included regardless of the amount of time elapsed between PCI and enrollment, though planned coronary intervention (such as PCI or coronary bypass surgery) was an exclusion criterion. Patients could be (re)evaluated for participation in the trial (starting with Visit 1.1) after their recovery from the intervention/surgery. Of note, randomization to icosapent ethyl versus placebo was stratified according to cardiovascular risk (established cardiovascular disease versus diabetes plus risk), geographic region, and ezetimibe use. In addition to prior PCI, all patients had been treated with a stable dose of statin for at least 4 weeks and had low‐density lipoprotein cholesterol under 100 mg/dL as well as serum triglycerides from 135–499 mg/dL. Other key inclusion and exclusion criteria for REDUCE‐IT have been published previously. All sites received ethics approval from relevant institutional review boards, and informed consent was obtained.

### Statistical Analysis

In this post hoc analysis, we analyzed patients enrolled in REDUCE‐IT who had a prior PCI. The primary and key secondary end points for this analysis were the same as the main REDUCE‐IT trial. The primary composite end point was the first occurrence of cardiovascular death, nonfatal myocardial infarction, nonfatal stroke, coronary revascularization, or unstable angina requiring hospitalization. The key secondary composite end point (or hard MACE end point) was the first occurrence of cardiovascular death, nonfatal myocardial infarction, or nonfatal stroke.

The intention‐to‐treat principle guided all analyses. Baseline characteristics were compared among groups using the Wilcoxon rank sum test for continuous variables and Chi‐square test for categorical variables. Hazard ratios (HRs) and 95% CIs were generated using Cox proportional‐hazard models that included risk stratum (established cardiovascular disease versus diabetes plus cardiovascular risk factors), geographic region, and ezetimibe use as covariates. It has been shown in other analyses that patients benefited from icosapent ethyl versus placebo regardless of baseline triglyceride levels, so this was not included as a covariable in this analysis. With Kaplan‐Meier analysis, we compared the time to events among patients randomized to icosapent ethyl versus placebo, with log‐rank *P* values also stratified by risk stratum, geographic region, and ezetimibe use.

As with other REDUCE‐IT analyses, we employed various statistical methods in comparing the risk for total (first and subsequent) events among patients treated with icosapent ethyl versus placebo.^17^ We used the negative binomial regression model to calculate rates and rate ratios (RRs) for total cardiovascular events. In supportive analyses, the modified Wei‐Lin‐Weissfeld method (Li and Lagakos modification taking into account death as a terminating event) was applied to calculate HRs for the time to the first and second, and a negative binomial model for rate ratios of third and greater events.^22^ As a sensitivity analysis, the Gray’s test was applied to the primary composite end point considering noncardiovascular death as a competing event. In addition to the primary and key secondary end points, results for additionally prespecified secondary end points in the original testing hierarchy are presented. Further post hoc explorations included time to total coronary revascularization and various revascularization subtypes (eg, elective, emergent, and urgent) as well as a coronary‐specific composite end point of myocardial infarction, coronary revascularization, or unstable angina. All statistical analyses were conducted using SAS 9.4 (SAS Institute, Cary, NC).

## Results

### Baseline Characteristics

Of the 8179 patients enrolled in REDUCE‐IT, 3408 (41.7%) had a prior PCI. In the 2559 patients with reported dates of PCI, the median time from PCI was 2.9 years, ranging from 11 days to 30.7 years. There were 675 (26.4%) patients with a PCI ≤1 year before randomization and 1884 (73.6%) with a PCI more than 1 year before randomization. Among patients in this study, the median age was 63 years, 20.7% were female, 96.3% were on moderate‐ or high‐intensity statin therapy, and the median triglyceride level was 218 mg/dL (Q1, Q3; 178.5 mg/dL, 274.5 mg/dL). There were no significant differences in baseline characteristics among patients randomized to icosapent ethyl versus placebo (Table 1).

**Table 1:** Baseline Characteristics

	Icosapent ethyl (N=1737)	Placebo (N=1671)	Overall (N=3408)	*P* value*
Age, y, median (Q1–Q3)^†^	63.0 (57.0–69.0)	63.0 (56.0–69.0)	63.0 (57.0–69.0)	0.73
Female sex, n (%)	350 (20.1)	354 (21.2)	704 (20.7)	0.46
White race, n (%)	1606 (92.5)	1539 (92.1)	3145 (92.3)	0.70
Westernized region, n (%)	1385 (79.7)	1313 (78.6)	2698 (79.2)	0.40
Cardiovascular risk category, n (%)				0.91
Established cardiovascular disease	1644 (94.6)	1583 (94.7)	3227 (94.7)	
Diabetes+risk factors	93 (5.4)	88 (5.3)	181 (5.3)	
Ezetimibe use, n (%)	138 (7.9)	150 (9.0)	288 (8.5)	0.28
Statin intensity, n (%)				0.22
Low	59 (3.4)	57 (3.4)	116 (3.4)	
Moderate	962 (55.4)	970 (58.0)	1932 (56.7)	
High	713 (41.0)	635 (38.0)	1348 (39.6)	
Missing	3 (0.2)	9 (0.5)	12 (0.4)	
Body mass index (kg/m^2^), median (Q1‐Q3)	30.5 (27.7–33.8)	30.3 (27.5–33.6)	30.4 (27.7–33.7)	0.32
Triglycerides (mg/dL), median (Q1‐Q3)	218.0 (180.5–271.5)	217.5 (177.5–277.0)	218.0 (178.5–274.5)	0.82
High‐density lipoprotein cholesterol (mg/dL), median (Q1‐Q3)	39.0 (34.0–45.0)	39.0 (34.0–45.5)	39.0 (34.0–45.5)	0.90
Low‐density lipoprotein cholesterol (mg/dL), median (Q1‐Q3)	73.0 (61.0–87.0)	74.0 (62.0–87.0)	74.0 (61.0–87.0)	0.37
Triglycerides category, n (%)				0.37
<150 mg/dL	154 (8.9)	167 (10.0)	321 (9.4)	
150 to <200 mg/dL	511 (29.4)	464 (27.8)	975 (28.6)	
≥200 mg/dL	1071 (61.7)	1040 (62.2)	2111 (61.9)	

* To assess balance between treatment groups, *P* values are reported from a Chi‐square test for categorical variables and Wilcoxon rank sum test for continuous variables. Missing categories are excluded from any comparisons.

^†^ Age (y) is at randomization.

### Clinical End Points

**Figure 1:**
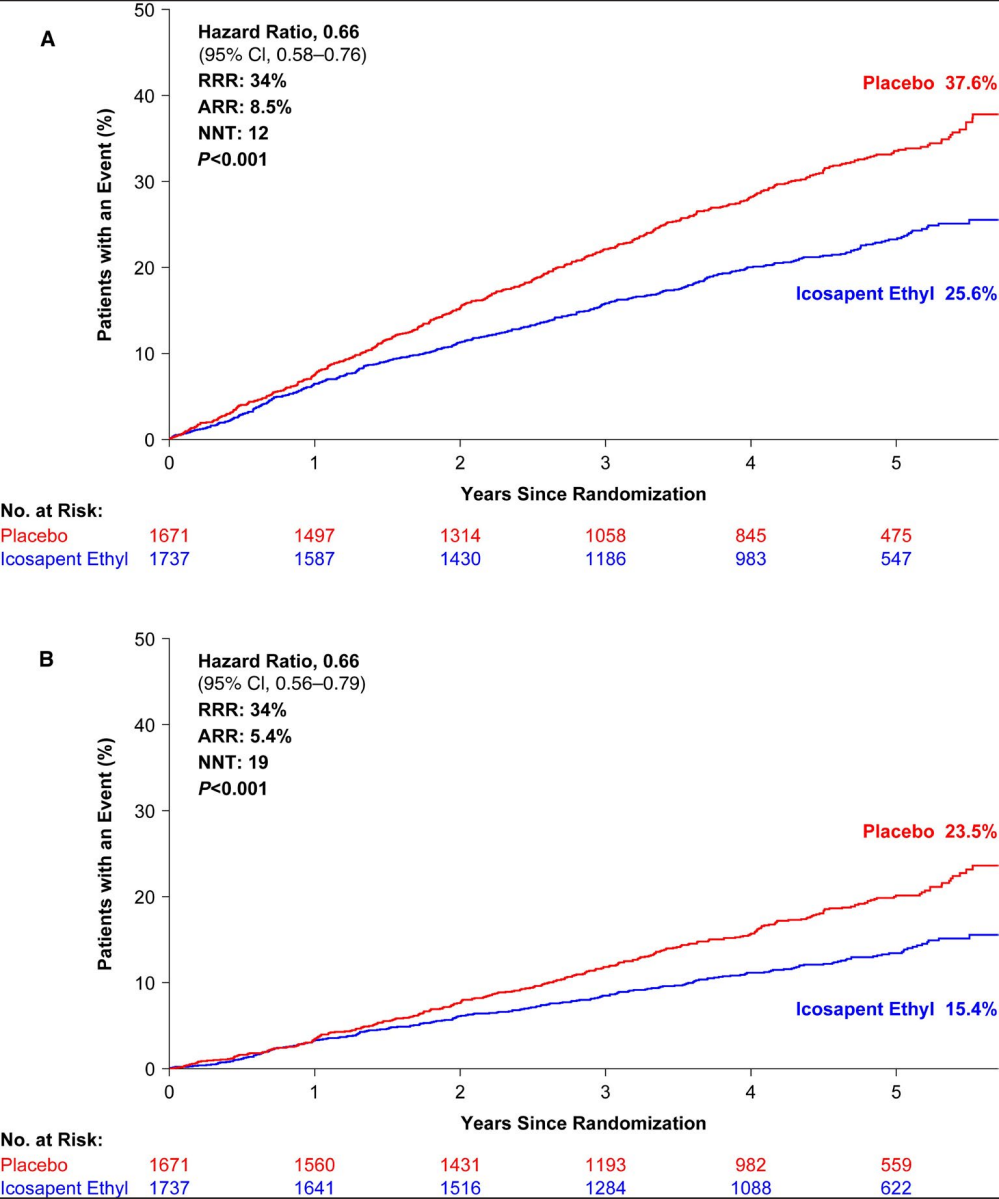
Kaplan‐Meier curves showing (**A**) time to primary composite end point (first cardiovascular death, myocardial infarction, stroke, coronary revascularization, or unstable angina requiring hospitalization) and (**B**) time to key secondary composite end point (first cardiovascular death, myocardial infarction, or stroke) among patients with prior percutaneous coronary intervention (PCI) treated with icosapent ethyl vs placebo. ARR indicates absolute risk reduction; NNT, number needed to treat; and RRR, relative risk reduction.

During a median follow‐up of 4.8 years, the rates of the primary composite end point (cardiovascular death, nonfatal myocardial infarction, nonfatal stroke, coronary revascularization, or unstable angina requiring hospitalization) were 20.8% among patients treated with icosapent ethyl and 29.4% among patients treated with placebo (HR, 0.66; 95% CI, 0.58–0.76; *P*<0.001). This represents a 34% relative risk reduction, an 8.5% absolute risk reduction, and a number needed to treat (NNT) of 12 patients to prevent 1 MACE event over a median of 4.8 years. The reduction in the primary end point with icosapent ethyl was similar in patients whose most recent PCI occurred ≤1 year before randomization (20.0% versus 29.7%, HR, 0.65; 95% CI, 0.48–0.89; *P*=0.007) and >1 year before randomization (20.3% versus 27.9%; HR, 0.68; 95% CI, 0.57–0.83; *P*<0.001). There was also a 34% reduction in the rate of the key secondary end point (cardiovascular death, nonfatal myocardial infarction, or nonfatal stroke) in patients treated with icosapent ethyl versus placebo (12.0% versus 17.4%, HR, 0.66; 95% CI, 0.56–0.79; *P*<0.001). The absolute risk reduction was 5.4%, NNT^4.8 years^=19 (Figure 1).

**Figure 2:**
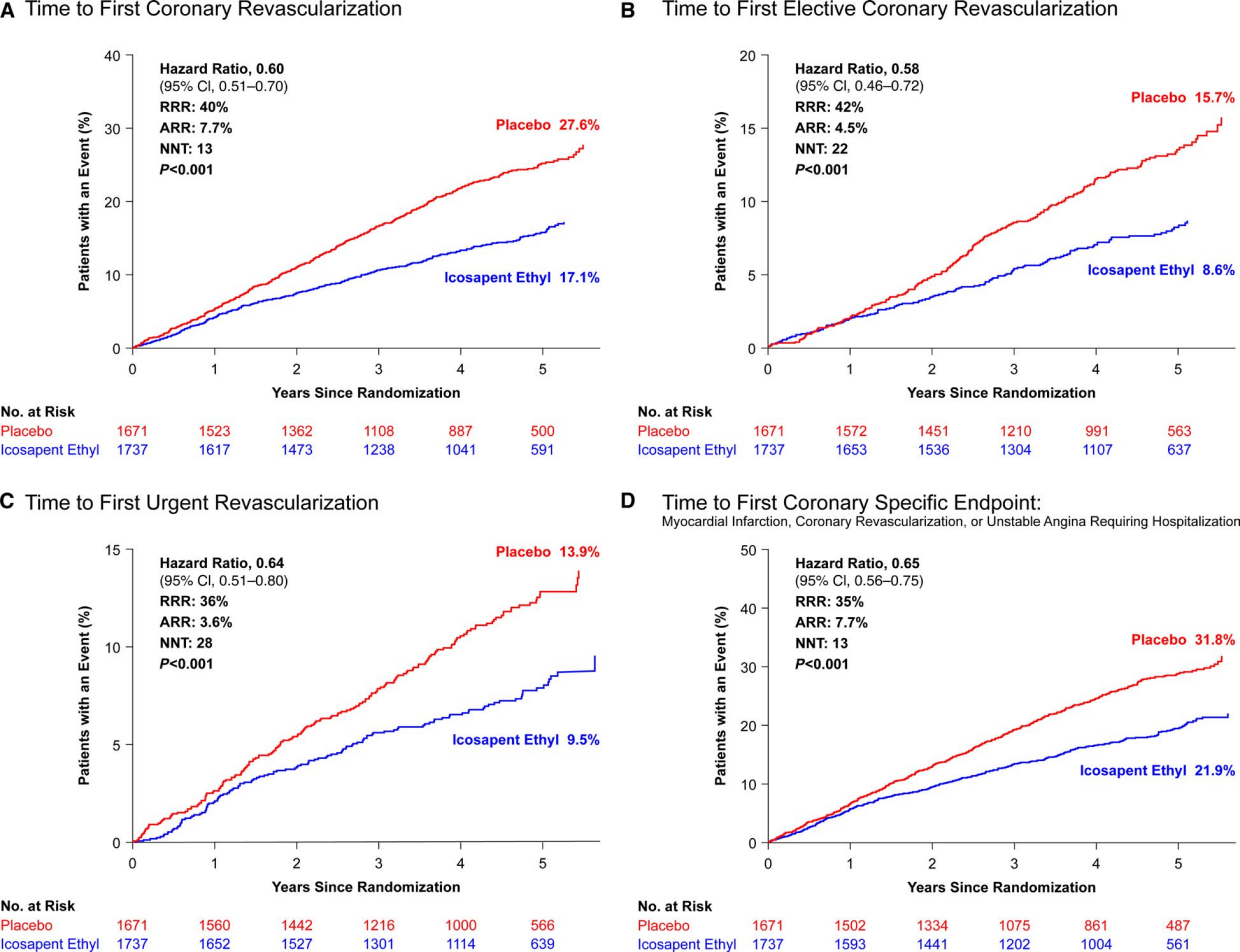
Kaplan‐Meier curves showing (**A**) time to first coronary revascularization, (**B**) time to first elective coronary revascularization, (**C**) time to first urgent revascularization, (**D**) time to first coronary specific end point: myocardial infarction, coronary revascularization, or unstable angina requiring hospitalization, among patients with prior percutaneous coronary intervention treated with icosapent ethyl vs placebo. ARR indicates absolute risk reduction; NNT, number needed to treat; and RRR, relative risk reduction.

Patients treated with icosapent ethyl experienced a significant 40% reduction in the risk of repeat coronary revascularization versus those treated with placebo (17.1% versus 27.6%; HR, 0.60; 95% CI, 0.51–0.70; *P*<0.001), with similar reductions in elective and urgent revascularization. There was also a significant reduction in the combined coronary end point of myocardial infarction, coronary revascularization, or unstable angina requiring hospitalization (HR, 0.65; 95% CI, 0.56–0.75; *P*<0.001) (Figure 2). There was no significant difference in the safety or efficacy of icosapent ethyl versus placebo among patients taking single‐ or dual‐antiplatelet therapy or a combined antithrombotic regimen (Figure S1). In addition, there were similar reductions in cardiovascular end points among women and men randomized to icosapent ethyl versus placebo (Figure S2).

**Figure 3:**
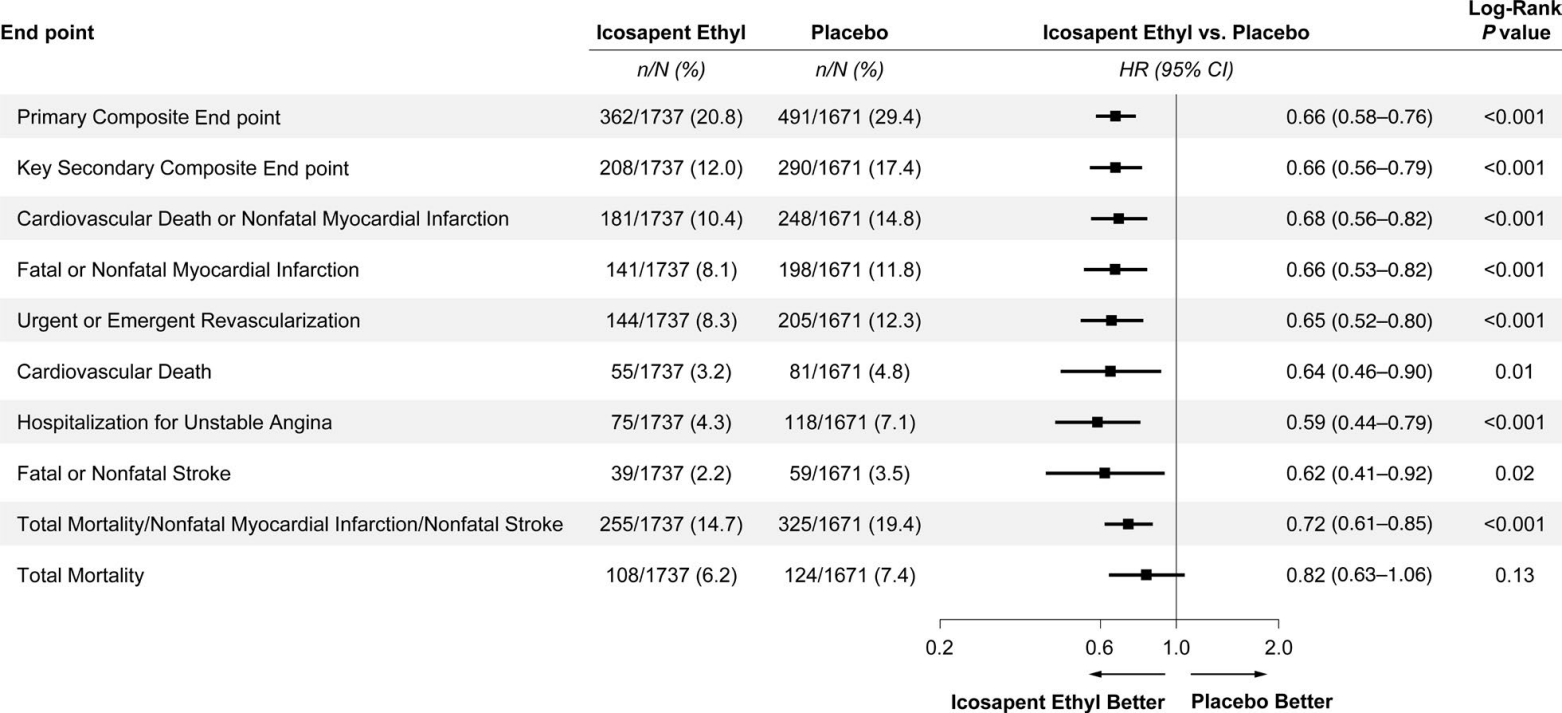
Hierarchical testing of end points: patients with prior percutaneous coronary intervention treated with icosapent ethyl vs placebo. HR indicates hazard ratio.

Testing in patients with prior PCI across the original prespecified hierarchical end points showed significant reductions in the primary and key secondary end points as well as in the following end points: cardiovascular death or nonfatal myocardial infarction; fatal or nonfatal myocardial infarction; urgent or emergent coronary revascularization; cardiovascular death; hospitalization for unstable angina; fatal or nonfatal stroke; and all‐cause mortality, myocardial infarction, or stroke (Figure 3). There were similar reductions in the primary and key secondary end points when accounting for noncardiovascular death as a competing risk factor (Figure S3). It should be noted that although the patients enrolled in REDUCE‐IT with cardiovascular risk factors and no history of PCI were a somewhat heterogeneous group, they had fewer cardiovascular events and derived a smaller in magnitude but still significant benefit from treatment with icosapent ethyl versus placebo (Figure S4).

### Total Events

**Figure 4:**
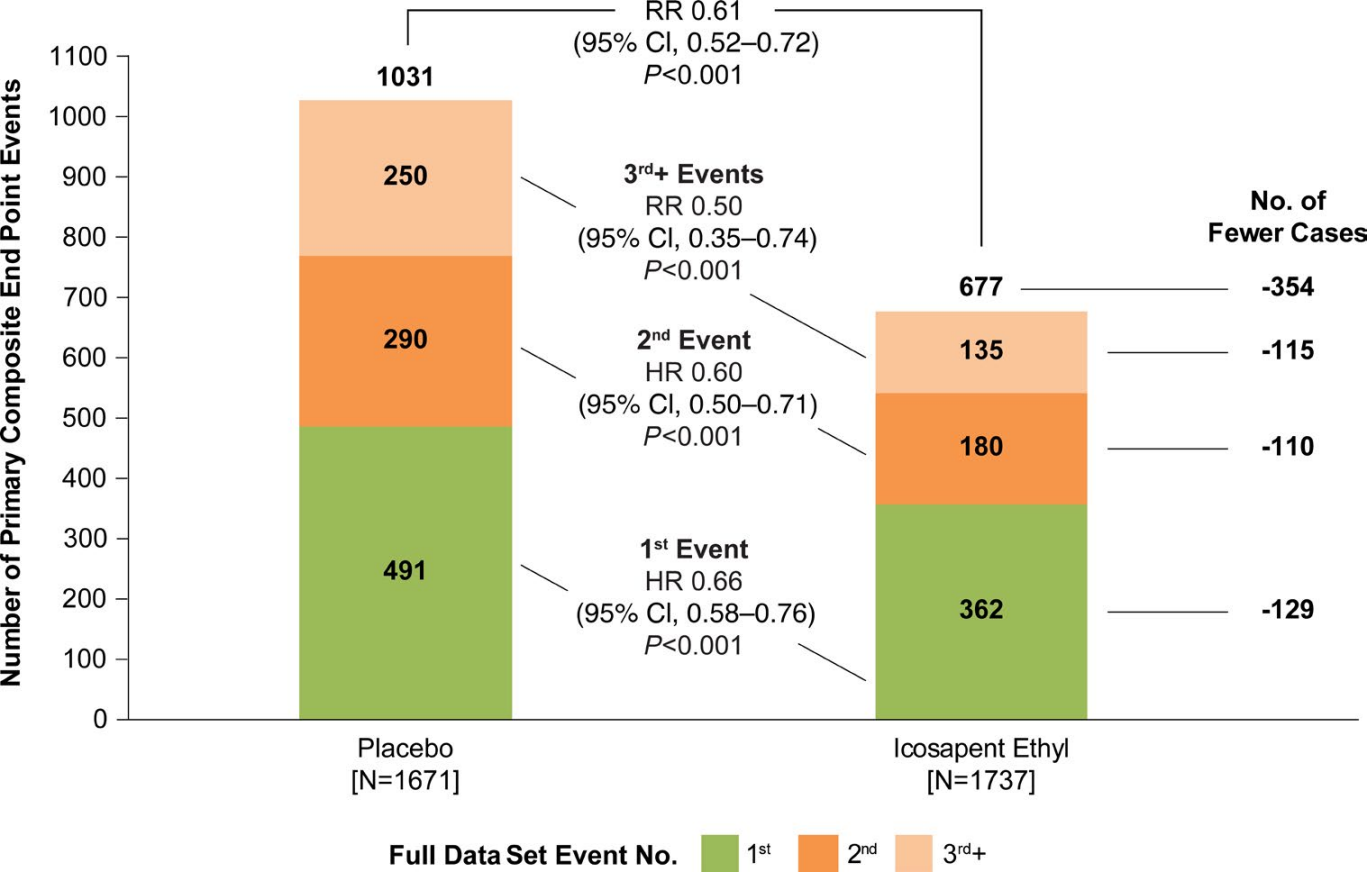
First, second, third or greater, and total events among patients with prior percutaneous coronary intervention treated with icosapent ethyl vs placebo. HR indicates hazard ratio; and RR, rate ratio.

Of the 1708 events that occurred during follow‐up, 853 (49.9%) were first events, 470 (27.5%) were second events, and 385 (22.5%) were third or greater events. During follow‐up, 1031 events occurred among patients treated with placebo and 677 events occurred among patients treated with icosapent ethyl. Using the negative binomial regression model, there was a significant 39% reduction in total (first and subsequent) events (RR, 0.61; 95% CI, 0.52–0.72; *P*<0.001) among patients treated with icosapent ethyl versus placebo. Icosapent ethyl also resulted in a significant 34% reduction in first events (HR, 0.66; 95% CI, 0.58–0.76; *P*<0.001), a significant 40% reduction in second events (HR, 0.60; 95% CI, 0.50–0.71; *P*<0.001), and a significant 50% reduction in third or greater events (HR, 0.50; 95% CI, 0.35–0.74; *P*<0.001) (Figure 4).

### Safety and Adverse Events

As in the primary REDUCE‐IT trial, among patients with a prior PCI treated with icosapent ethyl, there was a small increase in the number of patients who had adverse events of documented atrial fibrillation or flutter requiring emergency treatment (115 [6.6%] versus 75 [4.5%], *P*=0.007) or who had positively adjudicated end points of atrial fibrillation or flutter requiring hospitalization (59 [3.4%] versus 36 [2.2%]; *P*=0.04). There was no increase in total bleeding, any of the bleeding subtypes, or trial‐related adverse events (Table 2).

**Table 2:** Adverse Events

Adverse event, n (%)	Icosapent ethyl (N=1737)	Placebo (N=1671)	*P* value
Atrial fibrillation/flutter requiring emergency treatment*	115 (6.6)	75 (4.5)	0.007
Atrial fibrillation/flutter requiring hospitalization ≥24 hours^†^	59 (3.4)	36 (2.2)	0.04
Bleeding events+hemorrhagic stroke^‡^	226 (13.0)	205 (12.3)	0.54
Total bleeding events	221 (12.7)	202 (12.1)	0.60
Gastrointestinal bleeding	60 (3.5)	56 (3.4)	0.92
Central nervous system bleeding	13 (0.8)	7 (0.4)	0.26
Other bleeding	171 (9.8)	155 (9.3)	0.60
Hemorrhagic stroke	5 (0.3)	5 (0.3)	1.00
Severe TEAE	378 (21.8)	365 (21.8)	0.97
Serious TEAE	593 (34.1)	584 (34.9)	0.64

TEAE indicates treatment emergent adverse event.

* Includes atrial fibrillation/flutter TEAEs and excludes positively adjudicated events. *P* value is based on Fisher's Exact test.

^†^ Includes positively adjudicated atrial fibrillation/flutter requiring ≥24 hours of hospitalization clinical events by the Clinical Endpoint Committee. *P* value is based on stratified log‐rank test.

^‡^ Multiple bleeding TEAEs of the same preferred term are counted only once within each preferred term. Events that were positively adjudicated as clinical end points are not included in bleeding TEAEs. *P* values are based on Fisher's Exact test.

## Discussion

Among the 3408 patients in REDUCE‐IT with a prior PCI, icosapent ethyl taken 4 g daily (2 g twice daily) versus placebo resulted in a significant 34% reduction in the primary end point and a significant 34% reduction in the key secondary (hard MACE) end point. Even larger reductions occurred in second events, third or greater events, and total events. There were also significant reductions in total ischemic events, cardiovascular death, nonfatal myocardial infarction, nonfatal stroke, hospitalization for unstable angina, and coronary revascularization. The NNT^4.8 years^ to prevent 1 MACE event among patients treated with PCI over ~5 years was 12. In comparison, the NNT to prevent 1 MACE event was 22 over 5 years in the FRISC‐II (Framingham and Fast Revascularization During Instability in Coronary Artery Disease) trial,^23^ 50 at 7 years in IMPROVE‐IT (The Improved Reduction of Outcomes: Vytorin Efficacy International Trial),^24^ 67 at 2.2 years in FOURIER (Further Cardiovascular Outcomes Research with PCSK9 Inhibition in Subjects with Elevated Risk), 63 at 2.8 years in ODYSSEY OUTCOMES (Evaluation of Cardiovascular Outcomes After an Acute Coronary Syndrome During Treatment With Alirocumab).^25^, ^26^, ^27^

The patient population in this subgroup analysis of REDUCE‐IT reflects a large proportion of patients who undergo PCI in US, Canadian, and European registries.^28^, ^29^, ^30^, ^31^ Representative qualities include moderately elevated baseline triglycerides, with 50% of patients being <218 mg/dL and 96.3% on a moderate‐ or high‐intensity statin. The distribution of age, sex, and typical comorbidities also highly reflects contemporary populations undergoing PCI.^1^ Furthermore, this trial enrolled patients from November 2011 to August 2016 as the latest generation of drug‐eluting stents were employed, ameliorating the usual difficulty of interpreting clinical events in trials that had events during periods that used prior PCI technologies.^2^ Contemporary guidelines and consensus statements consistently recommend the use of icosapent ethyl in this patient population.^32^, ^33^

These findings of the overall REDUCE‐IT trial and this present analysis contrast sharply with neutral results from other contemporary clinical trials of moderate‐to high‐dose omega‐3 fatty acid supplementation, such as the recent STRENGTH (Long‐Term Outcomes Study to Assess Statin Residual Risk With Epanova in High Cardiovascular Risk Patients With Hypertriglyceridemia) and OMEMI (The Omega‐3 Fatty Acids in Elderly with Myocardial Infarction) trials.^34^, ^35^, ^36^ As well, older clinical trials have shown mixed results with respect to prevention of MACE events.^37^, ^38^, ^39^, ^40^, ^41^ In contrast, EPA in a highly purified form has shown a 19% benefit with respect to MACE in the open‐label JELIS (Japan EPA Lipid Intervention Study) at 1.8 g per day and a 25% reduction in MACE at 4 g per day in REDUCE‐IT.^11^, ^42^ In each of these trials, and even recently among patients with COVID‐19, high‐dose EPA in the form of icosapent ethyl has been well tolerated.^43^, ^44^

It may be that any benefit from omega‐3 fatty acid treatment is most directly tied to EPA alone, whereas other omega‐3 fatty acids, such as docosahexaenoic acid, may attenuate the benefit of EPA.^16^, ^45^ In REDUCE‐IT EPA, the on‐treatment levels of EPA correlated directly with reduction in the primary, key secondary, and individual cardiovascular end points.^46^ This suggests a dose‐dependent class effect specific to EPA rather than a broader impact associated with omega‐3 fatty acid supplementation alone. In REDUCE‐IT, the average patient taking icosapent ethyl experienced a 386% increase in serum EPA levels. Although some of the patients in STRENGTH receiving an EPA and docosahexaenoic acid mixture experienced a similar degree of elevation of EPA levels, they did not reap the same benefit.^34^ Further investigation may inform why this same benefit was not seen in the presence of simultaneous high‐dose docosahexaenoic acid supplementation. However, it is known that EPA and docosahexaenoic acid have very different tissue distribution and disparate effects on membrane stabilization and fluidity, formation of cholesterol rafts and crystals, rates of lipid oxidation in lipoproteins and cells, inflammatory modulation, transcriptional regulation, and endothelial function.^47^, ^48^, ^49^, ^50^, ^51^

Although the precise molecular mechanism of benefit from icosapent ethyl/EPA still requires some elucidation, the EVAPORATE (Effect of Vascepa on Improving Coronary Atherosclerosis in the People With High Triglycerides Taking Statin Therapy) trial has recently shed some important light on the gross vascular mechanism of benefit.^52^, ^53^ A total of 80 patients who had atherosclerotic coronary plaques with at least 20% stenosis on multidetector coronary computed tomography and a median baseline fasting triglyceride level of 259 mg/dL were randomized to icosapent ethyl 4 g daily versus placebo. Final follow‐up imaging at 18 months showed a significant 17% reduction in low‐attenuation plaque volume in patients treated with icosapent ethyl (whereas patients taking placebo nearly doubled their low‐attenuation plaque volume). Significant reductions also occurred in fibrofatty, fatty, total noncalcified, and total plaque volumes. Thus, high‐dose EPA therapy seems to result in significantly increased plaque stability and even plaque reduction, which could at least partially contribute to this marked reduction in cardiovascular events among high‐risk patients.

### Limitations

The exploratory nature as well as the lack of adjustment for multiple comparisons limited this post hoc analysis; REDUCE‐IT was not powered for this or other subgroup analyses. With the post hoc nature of these analyses, all *P* values should be considered hypothesis‐generating. As noted previously, patients with prior PCI had higher event rates and derived greater benefit from icosapent ethyl versus placebo. Although the time period from PCI to randomization was known in most patients, there was a subset of patients in which this was not known. Among the 24.9% of this subset and the remainder of patients, the distribution of randomization to icosapent ethyl versus placebo was equivalent. Double blinding also eliminated bias arising from this issue. The prerandomization extent of coronary artery disease and revascularization strategy (complete versus incomplete) among patients with a prior PCI was not known. Randomization was not stratified by history of PCI, and because there is potential for confounding, this subgroup finding needs corroboration in future studies. Future investigation will be required to gain a better understanding of whether icosapent ethyl reduced the rates of in‐stent restenosis versus de novo plaque events as vessel‐ and lesion‐specific data are not available in REDUCE‐IT. Also, had patients been enrolled soon after PCI when risk is highest, the degree of benefit seen here may have been even greater, especially if future studies validate the use of a loading dose.^43^

## Conclusions

Icosapent ethyl versus placebo resulted in significant and clinically meaningful reductions in cardiovascular events in this post hoc analysis. In patients with a prior PCI, the reductions in first and total primary end point events were 34% and 39%, respectively. There were large reductions in the primary and key secondary (hard MACE) end points, with NNTs^4.8 years^ of 12 and 19, respectively, and consistent benefit across the hierarchical end points. These data highlight the substantial positive impact of icosapent ethyl on patients in the REDUCE‐IT population, including patients with a history of prior PCI.

## Sources of Funding

The main REDUCE‐IT trial and this analysis have been funded by Amarin.

## Disclosures

Dr Bhatt serves as the Chair and International Principal Investigator for REDUCE‐IT, with research funding from Amarin to Brigham and Women’s Hospital. Dr Bhatt also discloses the following relationships—Advisory Board: Bayer, Boehringer Ingelheim, Cardax, CellProthera, Cereno Scientific, Elsevier Practice Update Cardiology, Janssen, Level Ex, Medscape Cardiology, MyoKardia, NirvaMed, Novo Nordisk, PhaseBio, PLx Pharma, Regado Biosciences, Stasys; Board of Directors: Boston VA Research Institute, DRS.LINQ (stock options), Society of Cardiovascular Patient Care, TobeSoft; Chair: Inaugural Chair, American Heart Association Quality Oversight Committee; Data Monitoring Committees: Acesion Pharma, Assistance Publique‐Hôpitaux de Paris, Baim Institute for Clinical Research (formerly Harvard Clinical Research Institute, for the PORTICO trial, funded by St. Jude Medical, now Abbott), Boston Scientific (Chair, PEITHO trial), Cleveland Clinic (including for the EXCEED trial, funded by Edwards), Contego Medical (Chair, PERFORMANCE 2), Duke Clinical Research Institute, Mayo Clinic, Mount Sinai School of Medicine (for the ENVISAGE trial, funded by Daiichi Sankyo; for the ABILITY‐DM trial, funded by Concept Medical), Novartis, Population Health Research Institute; Rutgers University (for the National Institutes of Health‐funded MINT Trial); Honoraria: American College of Cardiology (Senior Associate Editor, *Clinical Trials and News*, ACC.org; Chair, ACC Accreditation Oversight Committee), Arnold and Porter law firm (work related to Sanofi/Bristol‐Myers Squibb clopidogrel litigation), Baim Institute for Clinical Research (formerly Harvard Clinical Research Institute; RE‐DUAL PCI clinical trial steering committee funded by Boehringer Ingelheim; AEGIS‐II executive committee funded by CSL Behring), Belvoir Publications (Editor in Chief, *Harvard Heart Letter*), Canadian Medical and Surgical Knowledge Translation Research Group (clinical trial steering committees), Cowen and Company, Duke Clinical Research Institute (clinical trial steering committees, including for the PRONOUNCE trial, funded by Ferring Pharmaceuticals), HMP Global (Editor in Chief, *Journal of Invasive Cardiology*), *Journal of the American College of Cardiology* (Guest Editor; Associate Editor), K2P (Co‐Chair, interdisciplinary curriculum), Level Ex, Medtelligence/ReachMD (CME steering committees), MJH Life Sciences, Piper Sandler, Population Health Research Institute (for the COMPASS operations committee, publications committee, steering committee, and US national co‐leader, funded by Bayer), Slack Publications (Chief Medical Editor, Cardiology Today’s Intervention), Society of Cardiovascular Patient Care (Secretary/Treasurer), WebMD (CME steering committees); Other: *Clinical Cardiology* (Deputy Editor), NCDR‐ACTION Registry Steering Committee (Chair), VA CART Research and Publications Committee (Chair); Research Funding: Abbott, Afimmune, Aker Biomarine, Amarin, Amgen, AstraZeneca, Bayer, Beren, Boehringer Ingelheim, Bristol‐Myers Squibb, Cardax, CellProthera, Cereno Scientific, Chiesi, CSL Behring, Eisai, Ethicon, Faraday Pharmaceuticals, Ferring Pharmaceuticals, Forest Laboratories, Fractyl, Garmin, HLS Therapeutics, Idorsia, Ironwood, Ischemix, Janssen, Javelin, Lexicon, Lilly, Medtronic, Moderna, MyoKardia, NirvaMed, Novartis, Novo Nordisk, Owkin, Pfizer, PhaseBio, PLx Pharma, Recardio, Regeneron, Reid Hoffman Foundation, Roche, Sanofi, Stasys, Synaptic, The Medicines Company, 89Bio; Royalties: Elsevier (Editor, *Cardiovascular Intervention: A Companion to Braunwald’s Heart Disease*); Site Co‐Investigator: Abbott, Biotronik, Boston Scientific, CSI, St. Jude Medical (now Abbott), Philips, Svelte; Trustee: American College of Cardiology; Unfunded Research: FlowCo, Merck, Takeda. Dr Miller reports receiving consulting fees from Amarin and Akcea. Dr Brinton reports receiving fees as a speaker from Amarin, Amgen, Kowa, Regeneron, and Sanofi‐Aventis, and consulting fees from Akcea, Amarin, Amgen, Esperion, Kowa, Medicure, PTS Diagnostics, Regeneron, and Sanofi‐Aventis. Dr Jacobson reports receiving consulting fees from Amgen, Esperion, Novartis, Regeneron, and Sanofi. Dr Steg reports receiving research grant funding from Amarin, Bayer, Merck, Sanofi, and Servier; and speaking or consulting fees from Amarin, Amgen, AstraZeneca, Bayer/Janssen, Boehringer‐Ingelheim, Bristol‐Myers Squibb, Idorsia, Lilly, Merck, Novartis, Novo‐Nordisk, Pfizer, Regeneron, Sanofi, and Servier. Dr Ketchum, R.T. Doyle, Dr Juliano, Dr Jiao, and Dr Granowitz report being employed by and being stock shareholders of Amarin Pharma. Dr Tardif reports receiving grant support from AstraZeneca, Esperion, and Ionis, grant support and consulting fees from DalCor, grant support and fees for serving as co‐chairman of an executive committee from Pfizer, grant support and fees for serving on an executive committee from Sanofi, and grant support and consulting fees from Servier and holding a minor equity interest in DalCor and a patent (US 9 909 178 B2) on Dalcetrapib for Therapeutic Use. Dr Gibson reports research grant support and consulting fees from Amarin. Dr Pinto reports consulting fees from Abbott Vascular, Abiomed, Boston Scientific, Medtronic, Teleflex and consulting fees and stock options from NuPulseCV. Dr Giugliano reports that his institution received research grant support from Amgen, Bristol‐Myers Squibb, Merck, and The Medicines Company for clinical trials in lipid therapies, and honoraria for continuing medical education programs and/or consulting from Akcea, Amarin, Agmen, Bristol‐Myers Squibb, CVS Caremark, Daiichi Sankyo, GlaxoSmithKline, Merck, and Pfizer. Dr Budoff has received grant support and is on the speaker’s bureau for Amarin Pharmaceuticals. Dr Verma holds a Tier 1 Canada Research Chair in Cardiovascular Surgery; and reports receiving research grants and/or speaking honoraria from Amgen, AstraZeneca, Bayer, Boehringer Ingelheim, Bristol‐Myers Squibb, Eli Lilly, EOCI Pharmacomm Ltd, HLS Therapeutics, Janssen, Merck, Novartis, Novo Nordisk, Sanofi, Sun Pharmaceuticals, and the Toronto Knowledge Translation Working Group. He is the President of the Canadian Medical and Surgical Knowledge Translation Research Group, a federally incorporated not‐for‐profit physician organization. Dr Ballantyne reports receiving consulting fees from Arrowhead, AstraZeneca, Eli Lilly, Matinas BioPharma, Merck, Boehringer Ingelheim, Novo Nordisk, Denka Seiken, and Gilead and grant support (paid to his institution) and consulting fees from Amarin, Amgen, Esperion, Novartis, Regeneron, Sanofi‐Synthelabo, and Akcea. Dr Peterson has no disclosures to report.

## Supporting information

Appendix S1. REDUCE‐IT Investigators
Figures S1–S4
