# Supplementary material for: Treatment With Icosapent Ethyl to Reduce Ischemic Events in Patients With Prior Percutaneous Coronary Intervention: Insights From REDUCE‐IT PCI
Source: J Am Heart Assoc. 2022 Mar 9;11(6):e022937. doi: 10.1161/JAHA.121.022937 (PMC9075300; doi:10.1161/JAHA.121.022937)

Supplemental Material

Appendix S1. REDUCE-IT Trial Investigators

Steering Committee

Deepak L. Bhatt MD, MPH (Chair and Principal Investigator), Christie M. Ballantyne MD, Eliot A. Brinton MD, Terry A. Jacobson MD, Michael Miller MD, Ph. Gabriel Steg MD, Jean-Claude Tardif MD

Global Principal Investigator

Deepak L. Bhatt, MD, MPH, Professor of Medicine at Harvard Medical School, Executive Director of Interventional Cardiovascular Programs in the Heart and Vascular Center at Brigham and Women's Hospital, and the Principal Investigator and Steering Committee Chair for REDUCE-IT

The Netherlands National Coordinating Investigator

Fabrice MAC Martens, MD PhD, Board, Werkgroep Cardiologische centra Nederland (WCN; Dutch Network for Cardiovascular Research)

The Netherlands National Leader Office

Astrid Schut, MSc, Managing Director, Werkgroep Cardiologische centra Nederland (WCN; Dutch Network for Cardiovascular Research)

Data Monitoring Committee

Brian Olshansky MD (Chair), Mina Chung MD, Al Hallstrom PhD, Lesly Pearce MS (non-voting independent statistician)

Independent Statistical Center Support for Data Monitoring Committee

Cyrus Mehta PhD, Rajat Mukherjee PhD

Clinical Endpoint Committee

C Michael Gibson MD MS (Chair), Anjan K. Chakrabarti MD MPH, Eli V. Gelfand MD, Robert P. Giugliano MD SM, Megan Carroll Leary MD, Duane S. Pinto MD MPH, Yuri B. Pride MD

Amarin Operational and Statistical Team

Substantial Support Across the Study

Steven Ketchum PhD (President of R&D, Chief Scientific Officer, SVP) and team: Ramakrishna Bhavanthula MS, Gertrude Chester, Christina Copland PhD MPH, Katelyn Diffin MBA, Ralph Doyle Jr, Kurt Erz, Alex Giaquinto PhD, Paula Glanton MS, Angela Granger BA, Craig Granowitz MD PhD, Richard H Iroudayassamy BS, Lixia Jiao PhD, Rebecca Juliano PhD, James Jin PhD, Dimitry Klevak MS, Hardik Panchal MS, Robert Wang PhD, Shin-Ru Wang MS

Study Design and Initiation

Gerard Abate MD, Peggy J Berry MBA, Rene Braeckman PhD, Declan Doogan MD, Anne Elson, Amy HauptmannBaker, Isabel Lamela, Catherine Lubeck, Mehar Manku PhD, Sabina Murphy MPH, Monica Sanford, William Stirtan PhD, Paresh Soni MD PhD

Additional Operational and Statistical Support

Arnaud Bastien MD, Demetria Foster, Evangelito Gascon, Judith Johnson, Lasbert Latona MS, Gang Liu, PhD, Sandra Palleja MD, Nelly Sanjuan, Jimmy Shi MS, William Stager PhD, Mukund Venkatakrishnan MS, Ahmed Youssef-Agha PhD, Julie Zhu MD

Independent Statistical Support Center: Cytel, Inc.

Leela Aertker MS, Suresh Ankolekar PhD, Lisa Goldberg MS, Natasa Rajicic ScD, Jianfen Shu PhD, Heng Zou MS

Trial Operations

Bioclinica (data management)

Covance (central research laboratory)

Syneos Health™ (formerly inVentiv Health; principal contract research organization)

United States Investigators and Institutions:

United States Principal Investigators and Previous Principal Investigators

N=number of participants randomized; (site number)

N=3146 (100) Magdy Mikhail, (101) Gamil Dawood, (102) N. Mathew Koshy, (103) Sandip K. Mukherjee, (104) Rafik Abadier, (105) Andrea L. Lawless, (106) William P. McGuinn, (107) Howard Weintraub, (108) Kathryn Rohr, Edmund Claxton, Robert J. Weiss, (110) Terry D. Klein, (111) Mani Nallasivan, (114) Stephen Crowley, (115) Marilyn King, (116) Anthony D. Alfieri, (117) David Fitz-Patrick, (118) Irving Loh, (119) Nolan J. Mayer, (120) Rakesh Prashad, (121) Samuel Lederman, Debra Weinstein, (122) Harold E. Bays, (124) Keith Chu, Alireza Maghsoudi, (125) Paul D. Thompson, (129) Jeff Carstens, (130) Anna Chang, (131) Kenneth R. Cohen, (132) Julius Dean, (135) Howard S. Ellison, (136) Bernard Erickson, (137) Enrique A. Flores, (138) Daniel W. Gottlieb, (139) Paul Grena, (140) John R. Guyton, (141) Peter H. Jones, (142) John M. Joseph, (143) Norman E. Lepor, (144) Sam Lerman, (146) Robert D. Matheney, (147) Theodore R. Pacheco, (149) Michael B. Russo, (151) John Rubino, (152) Edward S. Pereira, Albert A. Seals, (154) Eduardo Viera, (155) Alan D. Steljes, (156) Jason Thompson, (158) Shaival Kapadia, (161) Michael McIvor, (162) Jorge E. Salazar, Jose O. Santiago, Ralph Vicari, (164) Martin R. Berk, (165) William A. Kaye, (166) Marcus McKenzie, (167) David Podlecki, (169) Brian D. Snyder, (171) Stephen Nash, (185) David M. Herrington, (186) Wallace Johnson, (189) Joseph R. Lee, Ronald Blonder, (190) Alpa M. Patel, Ramon Castello, Susan Greco, (191) Dean J. Kereiakes, (192) Venkatesh K. Nadar, (193) Mark Nathan, (194) Ranganatha P. Potu, (196) Robert Sangrigoli, (197) Richard Smalling, (199) Mitchell Davis, (203) Robert Braastad, James McCriskin, Kunal Bodiwala, (204) Joe L. Hargrove, (205) Mark W. Graves, (206) George Emlein, (207) Raegan W. Durant, (208) James W. Clower, (209) Rohit Arora, (211) Narendra Singh, (212) Lisa Warsinger Martin, (213) W Herbert Haught, (214) Marc P. Litt, (215) Michael D. Klein, (216) Peter Hoagland, (217) Michael Goldstein, (220) Marco S. Mazzella, Daniel H. Dunker, (221) Brian H. Kahn, Carlos S. Ince Jr., (222) Frank A. McGrew, (223) Jay Lee, David Pan, (224) Salman A. Khan, Uri Elkayam, (225) Wasim Deeb, (226) Anne C. Goldberg, (227) Christopher S. Brown, (228) Wayne N. Leimbach, (229) Thomas S. Backer, (230) David R. Sutton, (231) Matthew J. Budoff, (233) Joel Gellman, (234) Anu R. George, (235) Alan S. Hoffman, (237) Mark Kates, Kishlay Anand, Robert Bear, (239) Brendan J. Cavanaugh, (240) Ramon G. Reyes, (241) Rodolfo Sotolongo, (243) Kenneth Sabatino, (244) Kevin Gallagher, (246) Ehab Sorial, (248) Chris Geohas, (249) Kathleen E. Magness, (250) Bernard P. Grunstra, Frederik A. Martin, (251) William S. Knapp, Mel E. Lucas, (252) John J. Champlin, (253) Jason Demattia, (255) Patrick H. Peters Jr., (256) Judith Kirstein, (257) William J. Randall, (258) Cezar S. Staniloae, (259) Jennifer G. Robinson, (260) Alexander Adler, (261) Christopher Case, (263) Andrew J. Kaplan, (264) Gregory F. Lakin, Krishan K. Goyle, (265) Michael J. DiGiovanna, (267) Chester L. Fisher, (268) Michael Lillestol, (269) Michael Robinson, (270) Robert G. Perry, (272) Lawrence S. Levinson, (273) Brian G. Everhart, Robert D. Madder, (274) Earl F. Martin, Earl E. Martin, (275) Imtiaz Alam, (277) Jose Mari L. Elacion, (278) Robina Poonawala, (279) Taddese T. Desta, Jerome A. Robinson, (280) Gilbert J. Martinez, (281) Jakkidi S. Reddy, (283) Jeffrey D. Wayne, (284) Samuel Mujica Trenche, Westbrook I. Kaplan, Rubin H. Saavedra, Michael D. DiGregorio, (285) Barry D. Bertolet, (287)

Neil J. Fraser, (289) Terence T. Hart, (290) Ronald J. Graf, (291) David A. Jasper, Michael Dunn, (292) Dan A. Streja, (293) David J. Strobl, (295) Nan Jiang, (296) Vicki Kalen, (297) Richard Mascolo, (298) Mercedes B. Samson, (299) Michael Stephens, (350) Bret M. Bellard, (353) Mario Juarez, (356) Patrick J. McCarthy, (357) John B. Checton, (358) Michael Stillabower, Edward Goldenberg, (359) Amin H. Karim, (360) Naseem Jaffrani, (362) Robert C. Touchon, (363) Erich R. Fruehling, Clayton J. Friesen, Pradipta Chaudhuri, (364) Frank H. Morris, (365) Robert E. Broker, (367) Rajesh J. Patel, (368) Susan Hole, (370) Randall P. Miller, (371) Francisco G. Miranda, (373) Sadia Dar, (374) Shawn N. Gentry, (375) Paul Hermany, (376) Charles B. Treasure, (377) Miguel E. Trevino, (379) Raimundo Acosta, Anthony Japour, (380) Samuel J. Durr, (381) Thomas Wang, (383) Om P. Ganda, (384) Perry Krichmar, (386) James L. Arter, (387) Douglas Jacoby, (388) Michael A. Schwartz, (389) Amer Al-Karadsheh, (393) Nelson E. Gencheff, (394) John A. Pasquini, (396) Richard Dunbar, Sarah Kohnstamm, (398) Hector F. Lozano, (399) Francine K. Welty, (653) Thomas L. Pitts, (654) Brian Zehnder, (655) Salah El Hafi, (656) Mark A. King, (657) Arnold Ghitis, (661) Marwan M. Bahu, (662) Hooman Ranjbaran Jahromi, Ronald P. Caputo, (663) Robert S. Busch, (664) Michael D. Shapiro, (665) Suhail Zavaro, (668) Munib Daudjee, (669) Shahram Jacobs, (670) Vipul B. Shah, (671) Frank Rubalcava, (672) Mohsin T. Alhaddad, Henry Lui, (678) Raj T. Rajan, (679) Fadi E. Saba, (680) Mahendra Pai N Gunapooti, Tshiswaka B. Kayembe, (681) Timothy Jennings, (683) Robert A. Strzinek, (685) Michael H. Shanik, (686) Pradeep K. Singh, (687) Alastair C. Kennedy, (688) Howard Rubenstein, (690) Ramin Manshadi, (691) David M. Herrington

United States Sub Investigators / Study Coordinators and Site Staff
N=number of participants; (site number)

N=3146 (100) / Joanne Ladner, (101) / Lily Kakish, Ashley Kakish, (102) / Amy L. Little, (103) Jaime Gerber / Nancy J. Hinchion, Janet Guarino, (104) Denise Raychok / Susan Budzinski, (105) Kathleen Kelley-Garvin / April Beckord, Jessica Schlinder, (107) Arthur Schwartzbard / Stanley Cobos, (108) Deborah Freeman, David Abisalih, Dervilla McCann / Kylie Guy, Jennifer Chase, Stacey Samuelson, Madeline Cassidy, Marissa Tardif, Jaime Smith, (110) / Brenna Sprout, (111) Nanette Riedeman / Julie Goza, Lori Johnson, (114) Chad Kraske, Sheila Hastings / Chris Dutka, Stephanie Smith, Toni McCabe, (115) / Kathleen Maloney, (116) Paul Alfieri, Vinay Hosemane, Chanhsamone Syravanh, / (117) Cindy Pau, April Limcoiloc, Tabitha Carreira, Taryn S. Kurosawa / Taryn S. Kurosawa, (118) Razmig Krumian, Krista Preston, Ashraf Nashed, Daria Schneidman-Fernandez, Jack Patterson, John Tsakonas / Jennifer Esaki, Lynn Sprafka, Porous Patel, (119) / Brian Mitchell, Erin M. Ross, (120) / Donna Miller, Akash Prashad, (121) / Kristina M. Feyler, Natasha Juarbe, Sandra Herrera, (122) / Sarah M. Keiran, Becky Whitehead, Whitney Asher, Coury Hobbs, (124) / Abbey Elie, Jean Brooks, (125) / Amanda L. Zaleski, Brenda Foxen, (129) / Barb Lapke, (130) / Philippa Wright, (131) Bristol Pavol / Gwen Carangi, (132) / Marla Turner, (135) Howard S. Ellison, Katharine W. Sanders / Rikita S. Delamar, Virginia L. Wilson, Sarah M. Harvel, Alison M. Cartledge, Kaitlyn R. Bailey, (136) Kathleen Mahon, Timothy Schuchard / Jen Humbert, (137) Mark C. Hanson, Michael P. Cecil, James S. Abraham / Lorie Benedict, Claudia Slayton, (138) Curtis S. Burnett / Rachel W. Ono-Lim, (139) / Sharon Budzinski, (140) / Shubi A. Khan, (141) / Sharon Goss, Terry

Techmanski, (142) Farida Valliani / Rimla Joseph, (143) / Edith Flores, Laurn Contreras, Ana Aguillon, (144) / Carrie-Ann Silvia, Maria Martin, (146) Edmund K. Kerut, Leslie W. Levenson, Louis B. Glade, Brian J. Cospolich, Maureen W. Stein, Stephen P. LaGuardia, Thelma L. Sonza, Tracy M. Fife / Melissa Forschler, Jasmyne Watts, (147) / Judy Fritsch, (149) / Emese Futchko, (151) / Sarah Utech, (152) Scott B. Baker, Miguel F. Roura, David R. Sutton, Scott A. Segel, James S. Magee / Cathy Jackson, Rebecca F. Goldfaden, (154) / Liudmila Quas, (155) / Elizabeth C. Ortiz, (156) Michael Simpson, Robert Foster, Christopher Brian, James Trimm, Michael Bailey, Brian Snoddy, Van Reeder, Rachel Wilkinson, Harold Settle, Cynthia Massey, Angela Maiola, Michele Hall / Shelly Hall, Wanda Hall, (158) Mark Xenakis / Janet Barrett, (161) Giovanni Campanile, David Anthou / Susan F. Neill, (162) Steven Karas, Enrique Polanco, Norberto Schechtman / Grace Tischner, Kay Warren, Cynthia St Cyr, Menna Kuczinski, Latrina Alexander, (164) / Maricruz Ibarra, (165) Barry S. Horowitz, Jaime Steinsapir / Jeanette Mangual-Coughlin, Brittany Mooney, (166) / Precilia Vasquez, Kathleen Rodkey, Alexandria Biberstein, Christine Ignacio, (167) Irina Robinson / Marcia Hibberd, (169) Lisa B. Hoffman, Daniel J. Murak, Raghupathy Varavenkataraman / Theresa M. Ohlson Elliott, Linda A. Cunningham, Heather L. Palmerton, Sheri Poole, (171) / Jeannine Moore, Helene Wallace, (185) Ted Chandler, Robert Riley, Farah Dawood, Amir Azeem, Michael Cammarata, Ashleigh Owen, Shivani Aggarwal, Waqas Qureshi, Mohamed Almahmoud, Abdullahi Oseni, Adam Leigh, Erin Barnes, Adam Pflum, Amer Aladin / Karen Blinson, Vickie Wayne, Lynda Doomy, Michele Wall, Valerie Bitterman, (186) /Cindi Young, Rachel Grice, Lioubov Poliakova, (189) Jorge Davalos, David Rosenbaum, Mark Boulware, Heather Mazzola, J. Russell Strader, Russell Linsky, David Schwartz / Elizabeth Graf, Alicia Gneiting, Melissa Palmblad, Ashley Donlin, Emily Ensminger, Hillary Garcia, (190) Dawn Robinson, Carolyn Tran, Jeffrey Jacqmein, Darlene Bartilucci, Michael Koren / Barbara Maluchnik, Melissa Parks, Jennifer Miller, (191) / Cynthia DeFosse, (192) Albert B. Knouse, Jr / Amy Delancey, (193) Stephanie Chin, Thomas Stephens / Mag Sohal, Juana Ingram, (194) Swarooparani Kumar / Heather Foley, Nina Smith, (196) / Vera McKinney, Linda Schwarz, Judith Moore, (197) Hildreth Vernon Anderson, Stefano Sdringola-Maranga, Ali Denktas / Elizabeth Turrentine, Rhonda Patterson, (199) John Marshall / Terri Tolar, Donna Patrick, (203) / Pamela Schwartzkopf, (204) Anthony M. Fletcher, Frances R. Harris / Sherry Clements, Tiffany Brown, (205) William Smith / Stacey J. Baehl, Robin Fluty, (206) Daniel VanHamersveld, Dennis Breen, Nancy Bender / Beverly Stafford, Tamika Washington, (207) / Margaret N. Pike, (208) Mark A. Stich, / (209) Evyan Jawad, Amin Nadeem / Jill Nyland, (211) / Rhonda Hamer, Kendra Calhoun, Charlotte Mall, Samuel Cadogan, Kati Raynes, (212) Richard Katz / Lorraine Marshall, (213) Rashida Abbas, Jay L. Dinerman, John T. Hartley, III / Beth Lamb, Lisa Eskridge, Donna Raymond, (214) / Kristy Clemmer, (215) / Denise M. Fine, Paula Beardsley, (216) / Janet Werner, Bette Mahan, Courtney VanTol, (217) Robert Herman / Christine Raiser-Vignola, Felicia McShan, (220) Stefanie A. Neill, David R. Blick, Michael J. Liston / Denetta K. Nelson, Sandra K. Dorrell, Patricia Wyman, (221) Ambereen Quraishi, Fernando Ferro, Frank Morris / Vicki J. Coombs, Autumn M. Mains, Austin A. Campbell, Jeanne Phelps, Cheryl A. Geary, Ellen G. Sheridan, Jean M. Downing, (222) Arie Swatkowski, Tish Redden / Brian Dragutsky, Susan Thomas, Candace Mitchell, Diana Barker, Elanie Turcotte, Deborah Segerson, Jill Guy, (223) / Karena De La Mora, Jennifer Hong, Dennis Do, Rose Norris, (224) Faisal Khan / Hector Montero, (225) Stacy Kelly-White, Alan Cleland,

Rosalyn Alcalde-Crawford, Melissa Morgan, Brijmohan Sarabu, Megan Minor / Shweta Kamat, Stephanie M. Estes, Nancee Harless, Alicia Disney, (226) / Jodi L. Pagano, (227) Chad M. Alford, Noel W. Bedwell, Warren D. Hardy, Kevin DeAndrade / Jessica G. Elmore, (228) Eric G. Auerbach, Anthony W. Haney / Miriam H. Brooks, (229) Jose Torres, Lois Roper, Terry Backer, Katie Backer / Lois Roper, (230) John G. Evans, James S. Magee, Ricardo A. Silva, Lorraine H. Dajani, Scott A. Segel / Veronica Yousif, Tammy Ross, Rebecca F. Goldfaden, (231) Sion K. Roy, Ronald Oudiz / Sajad Hamal, Ferdinand Flores, (233) / Amor Leahy, Debra Ayer, (234) Swapna George / Chrisi Carine Stewart, Elvira Orellana, (235) Cristina Boccalandro / Mary Rangel, (237) Suzanne Hennings, Carl Vanselow, Teri Victor, Darlene Birdwell, Paul Haas, Anthony Sandoval / Gina Ciavarella, Caroline Saglam, (239) / Amy Bird, (240) Keith Beck, Brian Poliquin, David Dominguez, Brittany Tenorio / Harvonya Perkins, Esther San Roman, (241) Paris Bransford, Christy Lowrance, Marcy Broussard / Mary Ellis, Bobbi Skiles, Jessica Hamilton, Kathryn Hall, Diego Olvera, (243) / Kathleen Maloney, (244) / Julee A. Hartwell, (246) / Nevien Sorial, (248) Kishlay Anand, Mary Rickman, Kevin Berman, Nirav Mehta / Annie Laborin, Caroline Saglam, (249) Rodger Rothenberger / Sarah Beauvilliers, (250) / Kathy Morrell, (251) Michael P. Schachter, Cindy L. Perkins, Elizabeth A. Gordon / Jennifer Lauer, Kim Bichsel, Kelly Oliver, (252) / Leslie J. Mellor, (253) Candice Demattia / Jennifer Schomburg, Yenniffer Moreno, Eduardo Mansur-Garza, (255) Lena Rippstein / Lorie Chacon, Andrea Pena, (256) Michelle King / Susan Richardson, Annette Jessop, Nicole Tucker, Whitney Royer, (257) Gilbert Templeton, Ann Moell, Christine Weller / Melissa J. Botts, Gretel Hollon, (258) / Elsa Homberg-Pinassi, (259) Paula Forest, Aref Bin Abhulhak / Devona Chun-Furlong, Deborah Harrington, Emily Harlynn, Marjorie Schmitt, Constance Shelsky, Patricia Feldick, Mary Cherrico, (260) Courtney Jagle, Nicholas Warnecke, Debra Myer / Deanna J. Ruder, Albina Underwood, (261) Alan Rauba, George Carr / Barbara Oberhaus, Jessica Vanderfeltz, (263) Mary Jo Stucky-Heil, Dale R. Gibson / Vonnie Fuentes, (264) Kimberly L. Talbot, William C. Simon / Katlyn J. Grimes, Christina R. Wheeler, Cassaundra Shultz, Rhonda A. Metcalf, Jennifer L. Hill, Michelle R. Oliver, (265) / Basharat Ahmad, Fouzal Azeem, Abdul Rahim, (267) George H. Freeman, Dawn Bloch / Heather Freeman, (268) Jamie Brown / Sarah Rosbach, Pamela Melander, Nick Taralson, Alex Liu, Katlyn Harms, (269) Mahfouz Michale / Jose Lopez, (270) Maria Revoredo, / (272) / Shari Edevane, Sarah Shawley, (273) Timothy L. Jackson, Michael J. Oliver / Dina DeSalle, (274) Patricia J. Matlock, Ionna M. Beraun / Heather Hendrix, Garrett Bromley, Ashley Niemerski, Gabby Teran, Sonia Guerrero, Murtaza Marvi, Zehra Palanpurwala, Andrea Torres, Patty Gloyd, Michelle Conger, Andrea Pena, (275) Aziz Laurent / Olia Nayor, (277) Catalina S. Villanueva, (278) Munira Khambati / Tabetha J. Mumford, Melanie J. Castillo, (279) Taddese Desta, Jerome Robinson / La Shawn Woods, Anita H. Bahri, Nancy Herrera, (280) Cecilia Casaclang, Jeffrey R. Unger, Geraldine Martinez, Mia K. Moon, Stephen M. Mohaupt / Larry Sandoval, Louisito Valenzuela, Victora Ramirez, Nelly Mata, Veronica Avila, Marisol Patino, Cynthia Montano-Pereira, Omar Barnett, (281) William M. Webster / Lorraine M. Christensen, Leighna Bofman, Melanie Livingston, Stacey Adams, (283) Joseph Hobbs / Stacey Adams, Leesa Koskela, Mia Katz, (284) Samuel Mujica-Trenche / Franklin Cala, Noreen T. Rana, Jennifer Scarlett, Milagros Cala Anaya, (285) / Marsha R. Jones, Kelly D. Hollis, Debbie Roth, Kristin Eads, Tina Watts, (287) / Judy Perkins, (289) / Alice Arnold, (290) Daniel C. Ginsberg / Denise Quinn, (291) / Nicole Cureton, (292) David B.

Fittingoff, Mohammed I. Iqbal, Stephen R. White / Edith Sisneros, Michelle Ducca, David Streja, Danny Campos, (293) / Jennifer L. Boak, (295) / Farzeen Amir, (296) / Felice Anderson, (297) James J. Kmetzo / Mary O. Bongarzone, Dawn Scott, (298) / Mary Grace De Leon, (299) / Cynthia Buda, (350) William Graettinger, Michelle Alex, William Graettinger, / (353) Erika Hess, James Govoni / Melissa Bartel, (356) Travis L. Monchamp / Julie S. Roach, Sara Gibson, (357) / Amy M. Allfrey, Kristen Timpy, Kathy Bott, Karin A. Soucy, (358) / Jean Willis, (359) / Cecilia A. Valerio, Anusha Chunduri, (360) / Rebecca Coker, Nicole Vidrine, (362) Ellen A. Thompson, Mark A. Studeny / Melissa K. Marcum, Tammy S. Monway, (363) Douglas L. Kosmicki / Melissa J. Kelley, Corey M. Godfrey, Susan L. Krenk, Randy R. Holcomb, Deb K. Baehr, Mary K. Trauernicht, (364) David Rowland Lowry, Betty Bondy Herts, Jeanne E Phelps, Jean-Marie Downing, Carol Gamer Dignon, (365) / Elisabeth S. Cockrill, (367) Pravinchandra G. Chapla / Diane Fera, (368) Margaret Chang, Patricia Fredette / Tamie Ashby, Renee Bergin, (370) Zebediah A. Stearns, David B. Ware / Rachael M. Boudreaux, (371) Francisco G. Miranda / Joanna Rodriguez, (373) Robert McKenzie, Amanda Huber, Rebecca Sommers, Heather Rowe, Stacy McLallen, Michale Haynes / Ashley Adamson, Janice Henderson, Lori McClure, (374) / Beverly A. Harris, (375) / Laura Ference, Sue Meissner-Dengler, (376) / Lisa Treasure, Doreen Nicely, (377) Timothy L. Light / Tracey A. Osborn, Kimberly J. Mai, (379) Pablo Vivas, Jose Rios / Dunia Rodriguez, (380) Roger DeRaad, James Walder, Oscar Bailon / Denice Hockett, Debbie Anderson, Kelli McIntosh, Amber Odegard, Andrew Shepherd, Mary Seifert, (381) Laurence Kelley, Rajendra Shetty, Michael Castine, David Brill, Gregory Fisher / Nicole Richmond, Kathleen Gray, Patricia Miller, (383) / Charlene Coneys, (384) / Yarixa Chanza, Monica Sumoza, (386) Victoria M. Caudill, Kelly D. Harris, Courtney A. Manion / Melody J. Lineberger-Moore, Julie J. Wolfe, Barbara J. Rosen, (387) / Patricia DiVito, (388) / Janet L. Moffat, (389) Christina Michaelis, Prashant Koshy / Diana Perea, Ghaith Al Yacoub, Stephanie Sadeghi, (393) Thomas D. LeGalley, Rudolph F. Evonich, William J. Jean, Gary M. Friesen, John M. Pap, David A. Pesola, Mark D. Cowan, Kristofer M. Dosh / Dianna Larson, Adele M. Price, Jodi A. Nease, Jane E. Anderson, Lori A. Piggott, (394) Robert Iwaoka, Kevin Sharkey, Edward McMillan / Laurie Lowder, Latisha Morgan, Kyle Davis, Tara Caldwell, Erica Breglio, Jasmine Summers, Rachel Poulimas, (396) / Muhammad Zahid, Hamid Syed, Maria Escobar, Jacob Levy, Rahma Warsi, (398) Carol Ma, Puxiao Cen / Kimberly A. Cawthon, Delores B. Barnes, Deanna G. Allen, Margaret L. Warrington, Carol R. Stastny, Robin J. Michaels, (399) / Mohamad Saleh, (653) John Sorin / Sunny Rathod, Urakay Juett, (654) Steven Spencer, Aziza Keval, Jill McBride, Shane Young, Catherine Baxter, Carol Rasmussen, Shari L. Coxe / (655) Luis Campos, Shahin Tavackoli / Diana Beckham, Darlynee Sanchez, (656) Karanjit Basrai / Dorian Helms, Erica Clinton, Kasie Smith, (657) Arnold Ghitis, Henry Cusnir / Mary Klaus Clark, (661) Madhavagopal V. Cherukuri / Ameta Scarfaru, (662) Stephen D. Nash / Jeannine Moore, Helene Wallace, (663) Loretta C. Grimm, / (664) / Anna Grace, Kylie McElheran, (665) / Dino Subasic, (668) / Zedrick Buhay, (669) / Janet Litvinoff, Tamika Washington, (670) Deepak Shah / Shannon Cervantes, Freda Usher, (671) Farra Yasser, Theodore Trusevich, Ronnie L. Garcia, Jamison Wyatt, Rahul Bose / Holllilyn Miska, Traci Spivey, (672) Amy B. Wren, Katie E. Vance / Lani L. Holman, Pam Gibbons, (678) / Elaine Eby, Sandra Shepard, (679) Soratree Charoenthongtrakul, (680) / Brett Snodgrass, Mohammed Nazem, Shelly Keteenburg, Prathima Murthy, (681) Frederic Prater / Ashley Rumfelt, (683) / Christina

Eizensmits, (685) / Lisa Iannuzzi, (686) / Pourus R. Patel, (687) / Clellia Bergamino, Elizabeth McFeaters, (688) Botros Rizk, Emiljia Pflaum / Danny Kalish, (690) / Rex Ambatali, Mona Ameli, Delaina Sanguinetti / Mona Ameli, (691) Rakesh Vaidya / Karen Blinson, Lynda Doomy, Vickie Wayne

United States Institutions

N=number of participants randomized; (site number)

N=3146 (100) The Center for Clinical Trials, Inc., Biloxi, MS, (101) The Center for Clinical Trials, Mobile, AL, (102) Velella Research, Sarasota, FL, (103) Yale New Haven Health, North Haven, CT, (104) Nature Coast Clinical Research, Inverness, FL, (105) Biofortis, Inc, Addison, IL, (106) North Ohio Heart Center, Sandusky, OH, (107) New York University School of Medicine, New York, NY, (108) Maine Research Associates, Lewiston, ME, (110) Heartland Research Associates, LLC, Wichita, KS, (111) Merced Heart Associates, Merced, CA, (114) Aurora Denver Cardiology, Aurora, CO, (115) Clearwater Cardiovascular Consultants, Clearwater, FL, (116) Alfieri Cardiology, Wilmington, DE, (117) East-West Medical Research Institute, Honolulu, HI, (118) Westlake Medical Research, Westlake Village, CA, (119) Ventura Cardiology Consultants Medical Group, Inc., Ventura, CA, (120) Ocala Research Institute, Inc, Ocala, FL, (121) Altus Research, Inc, Lake Worth, FL, (122) L-Marc Research Center, Louisville, KY, (124) Carient Heart & Vascular, Manassas, VA, (125) Hartford Hospital, Hartford, CT, (129) Chi Health Research Center, Omaha, NE, (130) John Muir Physician Network Clinical Research Center, Concord, CA, (131) New West Physicians, Golden, CO, (132) St. Vincent's Research - Southside, Jacksonville, FL, (135) Rockdale Medical Research Associates, Conyers, GA, (136) Centracare Heart & Vascular Center, St. Cloud, MN, (137) Georgia Heart Specialists, LLC, Covington, GA, (138) Daniel W. Gottlieb, M.D., P.S., Burien, WA, (139) Cardiology Consultants of Philadelphia, Yardley, PA, (140) Duke University Medical Center, Durham, NC, (141) Baylor College of Medicine, Houston, TX, (142) Trinity Clinical Research Associates, Inc., Carrollton, TX, (143) Westside Medical Associates of Los Angeles, Beverly Hills, CA, (144) Jellinger and Lerman, MD PA Dba The Center for Diabetes and Endocrine Care, Fort Lauderdale, FL, (146) West Jefferson Heart Clinic of Louisiana, Marrero, LA, (147) North Ohio Heart Center, Lorain, OH, (149) Penn State Health Medical Group - Berks Cardiology, Wyomissing, PA, (151) PMG Research of Raleigh, Raleigh, NC, (152) East Coast Institute for Research, LLC, Jacksonville, FL, (154) Gables Research, Miami, FL, (155) Steljes Cardiology, Henderson, NV, (156) Birmingham Heart Clinic, Birmingham, AL, (158) Cardiovascular Associates of Virginia, Bon Secours St. Mary's Hospital, Midlothian, VA, (161) Foundation Research, Key West, FL, (162) Melbourne Internal Medicine Associates, Melbourne, FL, (164) Cardiovascular Research Institute of Dallas, Dallas, TX, (165) Metabolic Research Institute, Inc., West Palm Beach, FL, (166) Legacy Heart Center, Plano, TX, (167) Longmont Medical Research Network, Longmont, CO, (169) Southgate Medical Group, LLP, West Seneca, NY, (171) Syracuse Preventive Cardiology, Syracuse, NY, (185) Wake Forest University Health Sciences, Winston-Salem, NC, (186) University of Maryland School of Medicine, Baltimore, MD, (189) Memorial Hospital, University of Colorado Health, Colorado Springs, CO, (190) Jacksonville Center for Clinical Research Ltd, Jacksonville, FL, (191) The Lindner Research

Center, Cincinnati, OH, (192) Capital Area Research, Newport, PA, (193) University Healthcare Alliances/Cardiology Consultants Medical Group, Walnut Creek, CA, (194) Nature Coast Clinical Research, Crystal River, FL, (196) Doylestown Health Cardiology, Doylestown, PA, (197) University of Texas Health Science Center of Houston, Houston, TX, (199) Northeast Georgia Heart Center, Gainesville, GA, (203) Advocate Medical Group Cardiology/Pulmonology, Normal, IL, (204) Cardiology and Medicine Clinic, P.A., Little Rock, AR, (205) Research Institute of Deaconess Clinic, Evansville, IN, (206) Sacramento Heart and Vascular Research, Sacramento, CA, (207) University of Alabama at Birmingham, Birmingham, AL, (208) Westside Center for Clinical Research, Jacksonville, FL, (209) Captain James A. Lovell Federal Health Care Center, North Chicago, IL, (211) Atlanta Heart Specialists, LLC, Cumming, GA, (212) George Washington University School of Medicine and Health Sciences - Medical Faculty Associates, Washington, DC, (213) Heart Center Research, LLC, Huntsville, AL, (214) Baptist Heart Specialists, Jacksonville, FL, (215) Boston Medical Center, Boston, MA, (216) San Diego Cardiac Center, San Diego, CA, (217) Long Island Gastrointestinal Research Group LLP, Great Neck, NY, (220) Kansas City Cardiology, Lee's Summit, MA, (221) Spectrum Clinical Research at Overlea Personal Physicians, Baltimore, MD, (222) Stern Cardiovascular Foundation, Germantown, TN, (223) Orange County Heart Institute & Research Center, Orange, CA, (224) Metabolic Clinic and Research Center, Los Angeles, CA, (225) Baptist Endocrinology, Jacksonville, FL, (226) Washington University School of Medicine, St. Louis, MO, (227) Mobile Heart Specialists, Mobile, AL, (228) Oklahoma Heart Institute, Tulsa, OK, (229) Atlanta Cardiology Consultants, Roswell, GA, (230) East Coast Institute for Research, LLC, Jacksonville, FL, (231) Los Angeles Biomedical Research Institute at Harbor UCLA Medical Center, Torrance, CA, (233) Broward Health, Fort Lauderdale, FL, (234) Seven Corners Medical Research Center, Falls Church, VA, (235) Office of Dr. Alan S. Hoffman, Houston, TX, (237) Clinical Research Advantage Inc., Glendale, AZ, (239) New Mexico Heart Institute, PA, Albuquerque, NM, (240) BFHC Research, San Antonio, TX, (241) Southeast Texas Clinical Research Center, Beaumont, TX, (243) Clearwater Cardiovascular Consultants, Safety Harbor, FL, (244) United Medical Associates, Vestal, NY, (246) N & N Research and Management Corp., Fall River, MA, (248) Clinical Research Advantage, Phoenix, AZ, (249) PMA Medical Specialists, LLC, Phoenixville, PA, (250) PMG Research of Bristol, Bristol, TN, (251) Prime Care Research, LLC, Florissant, MS, (252) Med Center Medical Clinic, Carmichael, CA, (253) DM Clinical Research, Houston, TX, (255) Texas Medical Research Associates, LLC, San Antonio, TX, (256) Advanced Clinical Research, West Jordan, UT, (257) Primed Clinical Research, Dayton, OH, (258) NYU Langone Medical Associates Chelsea, New York, NY, (259) University of Iowa, College of Public Health, Preventive Intervention Center, Iowa City, IA, (260) Methodist Medical Center of Illinois, Peoria, IL, (261) Jefferson City Medical Group, P.C., Jefferson City, MS, (263) Cardiovascular Associates of Mesa, Mesa, AZ, (264) Professional Research Network of Kansas, LLC, Wichita, KS, (265) DiGiovanna Institute for Medical Education & Research, North Massapequa, NY, (267) Health Research of Hamilton Roads, Newport News, VA, (268) Lillestol Research LLC, Fargo, ND, (269) Clinica Medica San Miguel, Los Angeles, CA, (270) Pembroke Clinical Trials, Miami Lakes, FL, (272) Clinical Research Associates of Central PA, LLC, Altoona, PA, (273) Heritage Valley Medical Group, Inc., Beaver, PA, (274) Martin Diagnostic Clinic/DM Clinical Research, Tomball, TX, (275)

Austin Center for Clinical Research, Austin, TX, (277) Center for Clinical Trials, LLC, Paramount, CA, (278) Family Practice Center South, Austin, TX, (279) Precision Research Institute, San Diego, CA, (280) Catalina Research Institute, LLC, Montclair, CA, (281) Sierra Clinical Research, Roseville, CA, (283) Clinical Trials Research, Lincoln, CA, (284) Viable Research Management; Alas Science Clinical Research, Las Vegas, NV, (285) Cardiology Associates Research, LLC, Tupelo, MS, (287) Arcturus Healthcare, Plc, Troy Internal Medicine Research Division, Troy, MI, (289) Terence Hart MD, Tuscumbia, AL, (290) Multicare Research Institute, Tacoma, WA, (291) Quality Clinical Research, Omaha, NE, (292) Infosphere Clinical Research, INC., Omaha, NE, (293) Sparrow Clinical Research Institute, Lansing, MI, (295) Dairy Ashford Family Practice, Houston, TX, (296) Eclipse Clinical Research, Tucson, AZ, (297) Doylestown Health Cardiology, Doylestown, PA, (298) American Clinical Trials, Hawaiian Gardens, CA, (299) Fleming Island Center for Clinical Research, Fleming Island, FL, (350) Reno Clinical Trials, Sparks, NV, (353) Panacea Clinical Research, San Antonio, TX, (356) Endocrinology Services Northwest, Bend, OR, (357) Monmouth Cardiology Associates, Eatontown, NJ, (358) Christiana Care Health System, Newark, DE, (359) Angiocardiac Care of Texas, Houston, TX, (360) Alexandria Cardiology Clinic/Cambridge Medical Trials, Alexadria, LA, (362) Marshall Cardiology, Huntington, WV, (363) Nebraska Heart Institute, Hastings, NE, (364) Lutherville Personal Physicians, Lutherville, MD, (365) Hillcrest Clinical Research, LLC, Simpsonville, SC, (367) Lycoming Internal Medicine, Inc., Jersey Shore, PA, (368) Riverside Clinical Research, Edgewater, FL, (370) Horizon Research Group of Opelousas, LLC, Eunice, LA, (371) New Horizon Research Center, Miami, FL, (373) HCCA Clinical Research Solutions, Smryna, TN, (374) HCCA Clinical Research Solutions, Columbia, TN, (375) Grandview Lehigh Valley Health Services, Buxmont Cardiology Division, Sellersville, PA, (376) Cardiovascular Research of Knoxville, Knoxville, TN, (377) Innovative Research of West Florida, Clearwater, FL, (379) Research Physicians Network Alliance, Miami Beach, FL, (380) Black Hills Cardiovascular Research, Rapid City, SD, (381) Adventist Health Care Inc., Takoma Park, MD, (383) Joslin Diabetes Center, Boston, MA, (384) Research Physicians Network Alliance, Pembroke, FL, (386) CaroMont Heart & Vascular, Gastonia, NC, (387) Penn Presbyterian Medical Center, Philadelphia, PA, (388) Dupage Medical Group Cardiology, Winfield, IL, (389) Endocrine IPS, PLLC, Houston, TX, (393) UP Health System Marquette, Marquette, MI, (394) Novant Health Clinical Research, Charlotte, NC, (396) VA Medical Center, Philadelphia, Philadelphia, PA, (398) Florida Hospital, Orlando, FL, (399) Beth Israel Deaconess Medical Center, Boston, MA, (653) Diverse Clinical Research Center of Chicago, LLC, Chicago, IL, (654) Exodus Healthcare Network, Magna, UT, (655) Med-Tech LP, Houston, TX, (656) Trinity Medical Research, Inc, Roseville, CA, (657) Heart & Health Institute Westside, Plantation, FL, (661) Biltmore Cardiology, Phoenix, AZ, (662) SJH Cardiology, Liverpool, NY, (663) Albany Medical Center, Division of Community Endocrine, Albany, NY, (664) Oregon Health and Science University, Portland, OR, (665) Triwest Research Associates, El Cajon, CA, (668) Mercury Clinical Research, Inc, Houston, TX, (669) Shahram Jacobs, MD Inc, Sherman Oaks, CA, (670) Carolina Heart Specialists, LLC, Lancaster, SC, (671) Mission Research Institute, New Braunfels, TX, (672) Apex Cardiology, P.C., Jackson, TN, (678) Nova Clinical Research, Bradenton, FL, (679) Professional Health Care of Pinellas, St. Petersburg, FL, (680) Center for Advanced Medicine and Research, St. Peters, MO, (681) Clinical Research Professionals,

Chesterfield, MO, (683) Protenium Clinical Research, Hurst, TX, (685) Endocrine Associates of Long Island, PC, Smithtown, NY, (686) Synergist Research, LLC, Lancaster, CA, (687) Geodyssey Research, LLC, Vero Beach, FL, (688) The Center for Clinical Trials, Saraland, AL, (690) Manshadi Heart Institute, Inc, Stockton, CA, (691) W.G. (Bill) Hefner Salisbury VA Medical Center/ Kernersville Health Care Center, Kernersville, NC

Non-United States Investigators and Institutions:

Non-United States Principal Investigators and Previous Principal Investigators
Country listing by enrollment; N=number of participants randomized per country; (site number)

The Netherlands N=1678 ***Werkgroep Cardiologische centra Nederland (WCN)*** (701) Martinus AW Broeders, Dorman Henrikus, (702) Fabrice MAC Martens, (703) Adrianus FM Kuijper, (704) Nadea Al-Windy, (705) Michael Magro, Karim Hamraoui, (706) Ismail Aksoy, Guy L.J. Vermeiren, HWO Roeters van Lennep, (707) Gerard Hoedemaker, (708) Johannes Jacobus Remmen, (709) Kjell Bogaard, Dirk van der Heijden, (719) Nicole MJ Knufman, Joost Frederiks, (720) Johannes Willem Louwerenburg, (721) Piet van Rossum, Johannes Milhous, (722) Peter van der Meer, (723) Arno van der Weerdt, Rob Breedveld, (724) Mitran Keijzers, (725) Walter Hermans, (726) Ruud van de Wal, Peter AG Zwart, (727) Marc MJM van der Linden, Gerardus Zwiers, (728) Dirk J. Boswijk, Jan Geert Tans, (729) Jacob van Eck, (731) Maarten V. Hessen, (732) Barnabas JB Hamer, (733) Stieneke Zoet-Nugteren, (734) Lucien Theunissen, (735) EA van Beek, (736) Remco Nijmeijer, (737) Pieter R. Nierop, (738) Gerard Linssen, (739) H.P. Swart, (740) Timo Lenderink, (741) Gerard L. Bartels, (742) Frank den Hartog, (743) Brian J. Berg van den; ***non-WCN*** (710) Wouter van Kempen, (711) Susanne Kentgens, Gloria M. Rojas Lingan, Martinus M. Peeters, (712) Gloria M. Rojas Lingan, Hilligje Keterberg, Melchior Nierman, Annemieke K. den Hollander, (713) Jacqueline Hoogendijk, (714) Gloria M. Rojas Lingan, (715) Christine Voors-Pette, (716) Vicdan Kose, (718) Peter Viergever

Ukraine N=836 (885) Larysa Yena, (886) Viktor Syvolap, (887) Mykola P. Kopytsya, (888) Olga Barna, (889) Svitlana S. Panina, (890) Mykhailo I. Lutai, (891) Oxana V. Shershnyova, (892) Iryna Luzkiv, (893) Larysa S. Bula, (894) Sergii Zotov, Ivan Vyjhovaniuk, (895) Olena Lysunets, (897) Volodymyr I. Koshlia, (898) Nataliya Sydor, (899) Myroslava F. Vayda, (900) Olexiy Ushakov, (937) Mykola Rishko, (938) Viktor P. Shcherbak, (939) Yevgeniya Svyshchenko, (940) Vira Tseluyko, (941) Andriy Yagensky, (942) Viktoriia I. Zolotaikina, (943) Olga Godlevska, (944) Larysa Ivanova, (945) Olena Koval, (946) Olena I. Mitchenko, (947) Galyna Y. Kardash, (948) Yurii S. Rudyk, (949) Mykola Stanislavchuk, (951) Volodymyr Ivanovych Volkov, (952) Olena G. Karlinskaya, (953) Susanna A. Tykhonova, (954) Nikolay Vatutin, Ganna Smirnova, (955) Volodymyr M. Kovalenko, (956) Viktor Lizogub, (957) Denys Sebov, (958) Oleksandr Dyadyk, (959) Svetlana Andrievskaya, (960) Mykola P. Krasko, (961) Alexander N. Parkhomenko, (962) Lidiya Horbach, (963) Iryna G. Kupnovytska, (964) Tetyana Pertseva, (965) Oleksandr Karpenko, (985) Dmytro Reshotko, (992) Svitlana V. Zhurba, (994) Leonid Rudenko, (996) Viktoriia Yu Zharinova, (997) Valerii B. Shatylo, (998) Yuriy I. Karpenko, (999) Mariya A. Orynchak

Russian Federation N=709 (850) Tatiana R. Kameneva, (851) Elena Zherlitsina, (901) Diana N. Alpenidze, (902) Grigoriy P. Arutyunov, (903) Elena Baranova, (904) Boris Bart, (905)

Dmitriy I. Belenkiy, (906) Svetlana A. Boldueva, (908) Elena A. Demchenko, (909) Vera V. Eltishcheva, (910) Alexander M. Gofman, (911) Boris M. Goloshchekin, (912) Ivan Gennadyevich Gordeev, (913) Nikolay Gratsianskiy, (914) Gadel Kamalov, (915) Niyaz R. Khasanov, (916) Irina M. Kholina, (917) Zhanna D. Kobalava, (918) Elena V. Kobeleva, (919) Alexandra O. Konradi, (921) Victor A. Kostenko, (922) Andrey Dmitrievich Kuimov, (923) Polina Y. Ermakova, (924) Sofia K. Malyutina, (925) Alexey V. Panov, (926) Natalia V. Polezhaeva, (927) Olga Reshetko, (928) Nataliya P. Shilkina, (929) Sergey B. Shustov, (930) Elena A. Smolyarchuk, (931) Raisa I. Stryuk, (932) Elena Yurievnar Solovieva, Andrey V. Susekov, (933) Natalia Vezikova, (934) Svetlana N. Ivanova, (935) Alexander A. Petrov, (936) Vladimir O. Konstantinov, (966) Alina S. Agafina, (967) Victor Gurevich, (968) Konstantin N. Zrazhevskiy, (969) Tatiana V. Supryadkina, (970) Nikita B. Perepech, (971) Vadim L. Arkhipovskiy, (972) Dmitry Yu Butko, (973) Irina A. Zobenko, (974) Olga V. Orlikova, (975) Viktor Mordovin, (976) Olga L. Barbarash, (977) Anastasiya Lebedeva, (978) Vladimir Nosov, (979) Oleg V. Averkov, Elena P. Pavlikova, (980) Yuri B. Karpov, (982) Marina Lvovna Giorgadze, (983) Oleg A. Khrustalev, (987) Mikhail Arkhipov, (988) Tatiana A. Raskina, (989) Julia V. Shilko, (990) Yulia Samoilova, (991) Elena D. Kosmacheva, (993) Sergey V. Nedogoda

South Africa N=414 (416) Kathleen Coetzee, (417) Lesley J. Burgess, (418) FC R. Theron, (419) Iftikhar O. Ebrahim, (420) Gerbrand A. Haasbroek, (421) Maria Pretorius, (422) Julien S. Trokis, (423) Dorothea V. Urbach, (424) Mark J. Abelson, (425) Adrian R. Horak, (426) Aysha E. Badat, (427) Ellen M. Makotoko, Hendrik Du Toit Theron, (430) Padaruth Ramlachan, (431) Clive H. Corbett, (432) Ismail H. Mitha, (433) Hendrik FM Nortje, (435) Dirkie J. Jansen van Rensburg, (437) Peter J. Sebastian, (439) FC J. Bester, (440) Louis J. van Zyl, (441) Brian L. Rayner

Poland N=359 (602) Elżbieta Błach, (603) Magda Dąbrowska, (604) Grzegorz Kania, (605) Agata E. Kelm-Warchol, (606) Leszek P. Kinasz, (607) Janusz Korecki, (608) Mariusz Kruk, (609) Ewa Laskowska-Derlaga, (610) Andrzej Madej, (612) Krzysztof Saminski, (613) Katarzyna Wasilewska, (614) Katarzyna Szymkowiak, (616) Małgorzata Wojciechowska, (617) Natalia Piorowska, Andrzej Dyczek

India N=262 (501) Rajpal K. Abhaichand, (502) Ramesh B. Byrapaneni, (503) Basavanagowdappa Hattur, (504) Malipeddi Bhaskara Rao, (505) Nitin Ghaisas, Sujit Shankar Kadam, (506) Jugal B. Gupta, (507) Santhosh M. Jayadev, (509) V A. Kothiwale, (510) Atul Mathur, (511) Vijay Bhaskar, Ravi K. Aluri, Udaya P. Ponangi, (513) Mukesh K. Sarna, (514) Sunil Sathe, (515) Manish K. Sharma, Jilendra Pal Singh Sawhney, (516) Chakrabhavi B. Keshavamurthy, Arun Srinivas, (517) Hemant P. Thacker, (518) A Sharda, (524) Johny Joseph, (525) Sunil Dwivedi, (526) Viswanathan Mohan, (527) Rajendra K. Premchand

Canada N=250 (172) Jacques Bedard, (173) Jean Bergeron, (175) Ronald Collette, (176) David Crowley, (177) Richard Dumas, (178) Sam Henein, (181) Geoff Moran, (182) William F. O'Mahony, (188) Michael O'Mahony, (200) Sammy Chan, (201) Mark H. Sherman, (202) Graham C. Wong, (219) Brian D. Carlson, (271) Milan K. Gupta, David Borts, (361) Sean R. Peterson, Martyn Chilvers, (395) Allan J. Kelly, (397) Jean C. Gregoire, (659) Simon Kouz, (660) Josep Rodés Cabau

Romania N=202 (801) Minodora Andor, (803) Mircea Cinteza, (804) Radu Ciudin, (805) Radu I. Cojan, (806) Roxana O. Darabont, (808) Dan-Lucian Dumitrascu, (809) Carmen Fierbinteanu-Braticievici, (810) Ana Gabriela Fruntelata, (811) Constantin Militaru, (812) Bogdon E. Minescu, Doina Luminita Serban, (813) Florin Mitu, (814) Dorel Nastase Melicovici, (815) Ovidiu Petrascu, (816) Octavian M. Pirvu, (817) Cristian Podoleanu, (818) Calin Pop, (819) Rodica-Valentina V. Stanescu-Cioranu, (820) Adrian Tase, (821) Cristina Voiculet

Australia N=189 (301) Constantine N. Aroney, (302) Anthony M. Dart, (303) Timothy Davis, (304) Karam Kostner, (305) David N. O'Neal, (306) Peter W. Purnell, (307) Bhuwanendu B. Singh, (308) David R. Sullivan, (309) Peter Thompson, (310) Gerald F. Watts, (311) Adam F. Blenkhorn, (312) John V. Amerena, (313) Rafeeq Samie, Randall Hendriks, (314) Joseph Proietto, (316) Nikolai Petrovsky, (317) Alan Whelan, (319) David Colquhoun

New Zealand N=134 (402) Russell S. Scott, (405) Simon C. Young, (406) Tammy Pegg, Samuel JS Wilson, Andrew W. Hamer, (408) Richard A. Luke, (411) Hamish H. Hart, (414) Gerard P. Devlin, (415) Gerard T. Wilkins, (442) Ian F. Ternouth, (444) Samraj Nandra, Bruno S. Loeprich, Nicole McGrath, (445) Stuart L. Tie

Non-United States Sub Investigators / Study Coordinators and Site Staff
Country listing by enrollment; N=number of participants randomized per country; (site number)

The Netherlands N=1678 ***WCN*** (701) Rob J. Bos, Alexandra Wils / Tamara Jacobs, (702) Erik A. Badings / Lillian A. Ebels-Tuinbeek, Mayke L. Scholten, (703) / Esther Bayraktar-Verver, Debby Zweers, Manoek Schiks, Carolien Kalkman, (704) / Tineke Tiemes, Jeanette Mulderij, (705) Walter Hermans / Katarzyna Dabrowska, Wilma Wijnakker, Riny Van de Loo, Jeanne de Graauw, (706) / Giny Reijnierse, (707) / Mirjam van der Zeijst, (708) / Mariska Scholten, (709) / Henk R. Hofmeijer, Antoinette van Dijk-van der Zanden, (719) / Dineke J. van Belle, (720) Jan Van Es / Gera Van Buchem, Wendy Zijda, Harald Verheij, Linnea Oldenhof-Janssen, Martina Bader, Marije Löwik, (721) / Sandra Stuij, (722) Pascal Vantrimpont / Krista van Aken, Karen Hamilton, (723) Arno van der Weerdt / Han Blömer, Gabriela van Laerhoven, (724) Raymond Tukkie, Maarten Janssen, Gerard Verdel, Jon Funke Küpper, Bob van Vlies / Caroline Kalkman, Joke Vooges, Marinella Vermaas, (725) / Jeanne de Graauw, Riny Van de Loo, Rachel Langenberg, (726) Niek Haenen, Frans Smeets, Arko Scheepmaker, Marcel Grosfeld / Ilvy Van Lieshout, Marleen van den Berg, (727) / Marian Wittekoek, (728) / Petra Mol, Antionette Stapel, (729) Margaretha Sierevogel / Nancy van der Ven, Annemiek Berkelmans, (731) Eric Viergever / Hanneke Kramer, Wilma Engelen, Karen V. Houwelingen, (732) Thierry X. Wildbergh, Arend Mosterd / Coriet Hobé-Rap, Marjan van Doorn, Petra Bunschoten, (733) Michel Freericks, Mireille Emans / Petra Den Boer-Penning, Els Verlek, Christine Freericks, (734) Cornelis de Nooijer / Christina Welten, Ingrid Groenenberg, (735) / Claudia van der Horst, Esther Vonk, (736) Geert Tjeerdsma, Gerard M. Jochemsen / Corinne van Daalen, (737) / Ingrid Y. Danse, (738) / Lucy Kuipers, Anke Pieterse, (739) Antonius Oomen, Daan de Waard, Willem Jan Flu, Zusan Kromhout / Petra Van der Bij, (740) Rob Feld / Brigitta Hessels-Linnemeijer, Rob Lardinois, (741) Jan L. Posma / Zwanette R. Aukema-Wouda, Marjolijn Hendriks-van Woerden, (742) / Desiree van Wijk, (743) Driek P. Beelen / Ingrid H. Hendriks; ***non-WCN*** (710) Jan J. Jonker, Stefanie Schipperen, Vicdan Köse, Gloria Rojas /

Linda Goedhart, Hanneke van Meurs, Rachel Langenberg, Jacqueline Rijssemus, (711) Jacqueline Hoogendijk, Lindy Swinkels-Diepenmaat, Wouter van Kempen / Marloes de Louw-Jansen, Dominique Bierens-Peters, (712) Willem W. van Kempen, Marianne E. Wittekoek, Irmaina Agous / Geert Schenk, (713) Willem W. van Kempen, Janneke Wittekoek, Kevin Cox, Deborah F. Julia, Jan JC Jonker / Roel Janssen, (714) Willem W. van Kempen, Marianne E. Wittekoek, Melchor Nierman, Hilligje Katerberg, Jan JC Jonker / Irene van der Haar, (715) Willem W. Van Kempen, Taco van Mesdag, Janneke Wittekoek, Jan JC Jonker, Leyda M. Alvarez Costa / Manon Schensema, (716) Salomé Zweekhorst, Lindy Swinkels-Diepenmaat, Stefanie Schipperen, Willem W. van Kempen, Deborah Font Julia, Jan JC Jonker, Lauri Hanewinckel / Joyce Olsthoorn, (718) Johan C. Berends, Arie C. van der Spek, Roy van der Berg, Rob J. Timmermann / Ingrid Boerema

Ukraine N=836 (885) Iryna Mudruk, Anna Khrystoforova, / (886) Serhii Kyselov, / (887) Yaroslava V. Hilova, / (888) Pavlo Logoida / Pavlo Logoida, (889) Nataliia A. Sanina, / (890) Ilona P. Golikova, Olena O. Nemchyna / Ilona P. Golikova, Ilona P. Golikova, (891) Ivan I. Isaichikov, Olga B. Potapova / Iurii V. Gura, (892) Larysa Berestetska, / (893) Olena O. Kulianda, / (895) Oleksandr Tantsura, / (897) Oleksandr S. Kulbachuk, / (898) Volodymyr Petsentiy, Ihor Biskub / Ihor Biskub, (899) Tetyana Handych, (900) Oleg Lagkuti, Alyna Gagarina, / (937) Taras Chendey, / (938) Oksana F. Bilonko, / (939) Olena Matova, Larysa Bezrodna, Olena Yarynkina, Tetiana Ovdiienko, Volodymyr Randchenko, Maryna Mospan / Tetiana Ovdiienko, (940) Olena Butko, Olga Romanenko, / (941) Mykhailo Pavelko, Iryna Sichkaruk, / (942) Svitlana O. Lazareva, Olena A. Kudryk / Inessa M. Koltsun, Inessa M. Koltsun, (943) Tetiana Magdalits, / (944) Sergei Zadorozhniy, Kira Kompaniiets, / (945) Andrii Ivanov, Sergiy Romanenko, Pavlo Kaplan, / (946) Vadym Y. Romanov, / (947) Oksana P. Mykytyuk / Nataliia S. Zaitseva, (948) Sergiy N. Pyvovar, / (949) Lyudmyla Burdeuna, / (951) Emerita Serdobinska, / (952) Tatiana I. Shevchenko, Igor I. Ivanytskyi / Igor I. Ivanytskyi, Igor I. Ivanytskyi, (953) / Olena V. Khyzhnyak, (954) Ganna Smirnova, Nataliya Kalinkina, Olena Keting, Olena Sklyanna, Olga Kashanska, Anna Shevelok, Marina Khristichenko, / (955) Ievgenii Y. Titov, Danilenko O. Oleksander / Nataliia S. Polenova, (956) Nataliia Altunina, / (957) Viktoriia Kororaieva, / (958) Stanislav Zborovskiy, Leonid Kholopov, Iurii Suliman, Lanna Lukashenko, / (959) Stanislav Shvaykin, (960) Olexandr M. Glavatskiy, Roman O. Sychov, Roman L. Kulynych, / (961) Oleksandr A. Skarzhevskyi, Nataliia V. Dovgan, / (962) Marta Horbach, / (964) Olga Cherkasova, Iryna Tyshchenko, / (965) Liudmyla Todoriuk, Svitlana Kizim, Nataliia Brodi, Oleksandr Ivanko / Olga Garbarchuk, (985) Liudmyla Alieksieieva, / (992) Tetiana L. Shandra, / (994) Olena Beregova, / (996) Larisa An Bodretska, / (997) Svitlana S. Naskalova / Ivanna A. Antoniuk-Shcheglova, Olena V. Bondarenko, (998) / Natalia G. Andreeva, (999) Iryna I. Vakalyuk, Olha S. Chovganyuk, Nataliya R. Artemenko /

Russian Federation N=709 (850) Kiril A. Maltsev, / (851) Natalia Kalishevich, / (901) Natalia G. Kondratyeva, Svetlana A. Nikitina, Maria V. Martjanova, / (902) Anna V. Sokolova, Dmitrii O. Dragunov, / (903) Olga Kolesnik, / (904) / Vera Larina, (905) / Oxana V. Tsygankova, (906) Maria Ivanova, Illia A Karpov, Elena M Aronova, Ekaterina S. Vedernikova, / (908) / Ekaterina I. Lubinskaya, (909) Taras Y. Burak, / (910) Sergey I. Skichko, Farhad Rasulev / Ekaterina B. Soldatova, (911) Alexander L. Fenin / Ilya I. Laptev, (912) Elena E. Luchinkina, (913) Alexandr

Akatov, Natalia V Polenova, Natalia N Slavina, Irina N. Korovnika, Marina Yu Prochorova, / (914) Regina Shakirova, / (915) Elena N. Andreicheva, / (916) Olga A. Krasnova, / (917) Tinatin V. Lobzhanidze, Tatiana B. Dmitrova, / (918) Viktoriya V. Stakhiv, Maria I Pechatnikova, Alexandra V Panova, Maria Y. Tipikina, / (919) / Oxana P. Rotar, (921) Nikolay A. Bokovin, Saule K. Karabalieva, Farid Y. Tumarov / Elena V. Vasileva, (922) / Natalya Gennadevna Lozhkina, (923) Ekaterina V. Filippova, Alisa I. Sharkaeva / Ekanerina V. Filippova (Deilik), (924) Natalia Yu Tolkacheva, Elena N. Domracheva, Andrey N. Ryabikov, / (925) Inga T. Abesadze / Marianna Z. Alugishvili, (926) Elena P. Nikolaeva / Nadezda V. Smirnova, Valentina I. Rodionova, (927) Polina V. Dolovstaya, / (928) Igor E. Yunonin, / (929) Sergey V. Kadin, Tatyana S. Sveklina, / (930) Anna V. Bushmanova / Anna V. Bushmanova, (931) Elena L. Barkova, Irina S. Gomova, Yana V. Brytkova / Tatiana B. Ivanova, (932) Marina Y. Zubareva, / (933) Inga Skopets, / (934) Lybov A. Galashevskaya, / (935) Emilia D. Butinskaya / Olga G. Gusarova, (936) Natalia B. Kalishevich, Yana R Pavlova, Marianna P Serebrenitskaya, Vitalina F. Grygorieva, Gulnara R. Kuchaeva, / (966) Inna A. Vasileva, / (968) Gulnara I. Ospanova, (969) / Yulia V. Vahrusheva, Irina A. Semenova, (970) Irina E E. Mikhailova, Olga O. Kvasova, Valeria D. Shurygina, Alexey E. Rivin, Alexey O. Savelyev / Alexey A. Savelyev, (972) Olesya O. Milyaeva, Nadezhda N. Lapshina, Ninel A. Lantsova, / (973) Pavel V. Alexandrov, / (974) / Evgeniy A. Orlikov, (975) Alla Falkovskaya, Tatiana Ripp, Sergei Triss, Stanislav Pekarskiy / Sitkova Ekaterina, (976) / Evgeniya N. Zhuravleva, (977) Olga Perova, / (978) Galina Kovaleva, Liubov Koroleva / Liubov Koroleva, (979) Lydia Mishchenko, (980) Boris P. Garshin, / (982) Svetlana A. Kutuzova, Lyudmila I. Provotorova / Igor P. Zadvorny, (983) Olga V. Okhapkina / Anatoly O. Khrustalev, (987) Tatiana Suvorova, / (988) / Elena S. Shaf, (989) Varvara A. Vershinina, Andrey A. Kozulin, / (990) Oxana A. Oleynik / Irina Y. Martynova, (991) Natalia V. Kizhvatova, / (993) Alla S. Salasyuk, Vera V. Tsoma, Alla A. Ledyaeva, Elena V. Chumachek /

South Africa N=414 (416) SC Blignaut / Tersia Y. Alexander, Chano Du Plessis, (417) Thirumani Govender, Samatha M. Du Toit, Leya Motala / Areesh Gassiep, Christina Naude (Smit), Marli Terblanche, Marlien Snoer (Kruger), Berenice Pillay, (418) De Vries Basson, Clive H. Corbett / Marisa E. Theron, (419) / Bianca Fouche, Mareli E. Coetzee, (420) Pieter Odendall / Frederik H. Van Wijk, Anna-Mari Conradie, Trudie Van der Westhuizen, (421) / Carine Tredoux, (422) Mohamed S. Mookdam, Andie J. Van der Merwe / Karin Snyman, Gerda Smal, (423) / Yvonne De Jager, (424) Thomas A. Mabin / Annusca King, (425) / Lindy L. Henley, (426) / Brenda M. Zwane, Jane Robinson, (427) / Marinda Karsten, Andonia M. Page, Valerie Nsabiyumva, Charmaine Krahenbuhl, (430) Jaiprakash D. Patel, Yunus E. Motala / Ayesha Dawood, Nondumiso B. Koza, Lenore MS Peters, Shavashni Ramlachan, (431) Wilhelm J. Bodenstein, Pierre Roux / Lizelle Fouche, Cecilia M. Boshoff, (432) Haroon M. Mitha / Fathima Khan, (433) Henry P. Cyster / Helen Cyster, (435) E. C. Wessels / Florence J. Jacobs, (437) Melanie A. Sebastian / Deborah A. Sebastian, Nadia Mahomed, (439) Ignatius P. Immink / Celia Cotzee, (440) Tanja Cronje / Madele Roscher, Maria Le Roux, (441) Yvonne A. Trinder /

Poland N=359 (602) Renata Wnętrzak-Michalska / Magdalena Piszczek, (603) Andrzej Piela, Ewa Czernecka, Dorota Knychas, Alina Walczak, Izabella Gładysz / Katarzyna Filas, Ewelina Kiluk, Krzysztof Świgło, Iwona Jędrzejczyk, Kamila Łuczyńska, (604) / Katarzyna Tymendorf,

(605) Wojciech Piesiewicz, (606) Wojciech L. Kinasz / Stefan Samborski, Ilona Bartuś, (607) / Gramzyna Latocha Korecka, Ewa Gulaj, (608) / Jolanta Sopa, (609) Bogusław Derlaga, / (610) Marcin Baisiak, / (612) Allicia Kowalisko, Edyta Stainszewska-Marasazlek, Bartosz Szafran / Malgorzata Swiatkiewicz, (613) Artur Racewicz, Sławomir Grycel, Jerzy Supronik / Sylwia Walendziuk, Magdalena Tarantowicz, Agata Stasiak, (614) Anna Sidorowicz-Białynicka, Marek Dwojak, Ewa Jaźwińska-Tarnawska / Katarzyna Kupczyk, Kamila Martowska, Kamila Kulon, (617) / Katarzyna Gajda

India N=262 (501) Bivin Wilson / Krithika Velusamy, Swaidha S. Sadhiq, (502) / Bhavani Siddeshi, (503) M Bhanukumar / Abhishek Srivatsav, Madhan Ramesh, Sri Harsha Chalasani, Mini Johnson, Prashanth Gopu, Jeesa George, Sowmya Reddy, Swetha Tessy Thara Eleena (504) Damodara Rao Kodem / Haritha N. Nakkella, Padma Kumari Mandula, Anjan Kumar Vuriya, Syamala Rajana, (505) / Aruna Kale, (506) Tiwari Rajeev / Raina Jain, Vipin Jain, (507) Srilakshmi Mandayam Adhyapak / Lumin Sheeba, Uma C R, Ramya R, (509) Aditya V. Kulkarni / M S. Ganachari, Ruma Sambrekar, (510) / Mohammad Bilal, Nungshijungla (511) Kalyan Chakravarthy / Ravi Badhavath, Sravan Kumar, Meenakshi Simhadri, Farooque Salamuddin, Venkat Prasad, (513) Vivek Dwivedi, Sudha Sarna / Tilak Arora, Deepak Chawla, (514) Archana Sathe / Chaware Gayatree, (515) / Ajeet Nanda, Ram Avtar, Jyoti Sharma, (516) Vaibhavi P S Sasirekha D, Deepthi Kobbajji / Ramya Ningappa, Shwetha Shree, Chandrashekar K Nandini M R Sowjanya S Devika I G Yashaswini N Sonika G Rathna L Priyanka R (517) / Rupal J. Shrimanker, (518) Lakshmi Vinutha Reddy, K Sumathi, Babitha Devi / Bina N. Naik, Rohini Manjunath, Rajeshwari Ashok, (524) / Tony V. Kunjumon, Jesline Thomas, (525) / Shaik Samdhani, (526) Kasthuri Selvam / Poongothai Subramani, Nandakumar Parthasarathy, (527) Nirmal K. Bohra / Anvesh K. Gatla

Canada N=250 (172) / Cheryl Horbatuk, (173) / Julie Sills, (175) E B. Davey / Liz Paramonczyk, Olga Racanelli, (176) David Crowley / Sandy Strybosch, (177) Andre Belanger, Jean Palardy, Alicia Schiffrin / Sylvie Gauthier, (178) Norman Kalyniuk, Shawn D. Whatley / Heather Lappala, Grishma Patel, Matthew Reeve, (181) Catherine Moran / Jody Everitt, (182) / Teresa Ferrari, (188) / Christine Bouffard, (200) Jirir Frohlich, Gordon Francis, John Mancini, Gregory Bondy, Debbie DeAngelis, Patricia Fulton / Debbie DeAngelis, Patricia Fulton, (201) David W. Blank / Angela Lombardo, Mylène Roy, (202) / Jackie Chow, (219) Hyman Fox, William J. Grootendorst, Angela Hutchinson, Hyman Fox / Sharon M. Chan, (271) / Christie Fitzgerald, (361) / Teresa Ferrari, (395) / Lynn Wilkins, Rebecca L. Raymond, Arlene Reyes (397) Lavoie Marc André / Denis Fortin, (659) Hélène Ouimet, Thanh-Thao Tôn-Nu, Martine Dussureault, Marie-Hélène Blain / Madeleine Roy, Nathalie Kopajko, Chantal Fleury, (660) / Karine Maheux

Romania N=202 (801) Gabriela Valentina Ciobotaru, / (803) Maria C. Constantinescu / Carmen-Lucia Gherghinescu, (804) Ana-Maria Avram, / (805) Ioan Manitiu / Radu I. Cojan, (806) Octavian M. Pirvu, (808) Aura Sinpetrean, Lucian Pop, Delia Lupu, / (809) Radu Usvat, Ana Petrisor, / (810) Nicoleta Dumitru, / (811) Camelia Moruju, / (812) / Adelina Gheorghita, (813) Magda V. Mitu, / (814) Cosmin Macarie, / (815) Ana Maria Pop, / (816) Maria-Catalina Diaconu, / (817) Iulia Grancea, / (818) Mihaela Cosma / Mihaela Cosma, (819) Mihaela Crisan /

Australia N=189 (301) / Elizabeth Herron, (302) Anthony M. Dart, Paul Nestel / Sally B. Kay, Kaye S. Carter, (303) Imran Badshah, Ashley Makepeace / Jocelyn Drinkwater, Michelle England, (304) / Azette Rafei, Kylie Patterson, (305) Alicia Jenkins, Sybil McAuley / Sue M. Kent, (306) / Joy E. Vibert, Leonie Perrett, (307) Thomas David / Samantha L. Kaye, Monika O'Connor, (308) Nimalie J. Perera / Nicole T. Lai, Kerry A. Kearins, (309) Christinia Dicamillo, Heather Anderson / Louise Ferguson, (310) / Sharon D. Radtke, (311) Charles T. Thamarappillil / Janice M. Boys, (312) / Anita K. Long, Toni Shanahan, (313) Michael Nyguyen / Nicole Forrest, Gill Tulloch, Della Greenwell, (314) Sarah L. Price, Aye N. Tint, Priya K. Sumithran / Tamara L. Debreceni, Lisa Walker, Mary Caruana, Kira Edwards, Maria Stathopoulos, Cilla Haywood, (316) Dimitar Sajkov / Sharen Pringle, Anne Tabner, Kathrina Bartolay, Chamindi Abeyratne, Kylie Bragg, (317) Patrick Mulhern, Peter Purnell, Randall Hendriks / Gill Tulloch, (319) Lyn Williams, Jane Hamlyn / Aurelia Connelly, Jan Hoffman

New Zealand N=134 (402) Samantha Bailey, Jane Kerr / Zarnia Morrison, Sarah Maeder, Roberta McEwan, Prasanna Kunasekera, Patrice McGregor, Jo Young, Sharon Berry, (405) Rick Cutfield, Michelle Choe, Catherine McNamara / Narrinder K. Shergill, (406) / Petra Crone, (408) Miles G. Williams, Keith Dyson / Diana H. Schmid, Audrey C. Doak, Melissa Spooner, (411) Colin Edwards / Anne Turner, Grainne M. McAnnalley, (414) Raewyn A. Fisher, Fraser B. Hamilton, Denis H. Friedlander / Melissa R. Kirk, Jayne E. Scales, (415) / Marguerite A. McLelland, (442) Neelam A. Dalman / Cathy E. Vickers, Carolyn Jackson, (444) / Wendy Coleman, (445) Phillip I. Garden / Wendy F. Arnold

Non-United States Institutions
Country listing by enrollment; N=number of participants randomized per country; (site number)

The Netherlands N=1678 ***WCN*** (701) Bravis Hospital, Roosendaal, (702) Deventer Hospital, Cardiology Department, Deventer, (703) Spaarnegasthuis, Hoofddorp, (704) Gelre Ziekenhuis, Zutphen, (705) Tweesteden Ziekenhuis, Tilburg, (706) Admiraal De Ruyter Ziekenhuis, Goes, (707) Tergooi, Blaricum, (708) Canisius Wilhelmina Ziekenhuis, Nijmegen, (709) Alrijne Hospital, Leiderdorp, (719) HMC Bronovo, Den Haag, (720) Stichting CRE Enschede, Thoraxcentrum Twente, Medisch Spectrum Twente, Enschede, (721) Beatrix Hospital, Gorinchem, (722) Langeland Ziekenhuis, Cardiology Department, Zoetermeer, (723) Medisch Centrum Leeuwarden, Leeuwarden, (724) Spaarne Gasthuis, Haarlem, (725) St Elisabeth Hospital, Tilburg, (726) Bernhoven Hospital, Uden, (727) Franciscus Gasthuis & Vlietland, Schiedam, (728) Noordwest Ziekenhuis, Den Helder, (729) Jeroen Bosch Hospital, Hertogenbosch, (731) Groene Hart Ziekenhuis, Gouda, (732) Meander Medical Center, Amersfoort, (733) Ikazia Hospital Rotterdam, Rotterdam, (734) Máxima Medisch Centrum, Veldhoven, (735) Ziekenhuis Stjansdal, Harderwijk, (736) Tjongerschans Ziekenhuis, Cardiology Department, Heerenveen, (737) Franciscus Gasthuis, Rotterdam, (738) ZGT, Almelo and Hengelo, (739) D & A Research, Sneek, (740) Zuyderland Mc, Heerlen, (741) Martini Ziekenhuis, Groningen, (742) Gelderse Vallei Ziekenhuis, EDE, (743) Ijsselland Ziekenhuis, Capelle aan den Ijssel; ***non-WCN*** (710) Andromed Rotterdam, Rotterdam, (711) Andromed Eindhoven, Eindhoven, (712) Andromed Leiden, Leiderdorp, (713) Andromed Oost BV, Velp,

(714) Andromed Zoetermeer BV, Zoetermeer, (715) Andromed Noord, Groningen, (716) Andromed Breda, Breda, (718) Gemini Ziekenhuis, Den Helder

Ukraine N=836 (885) State Institutio, D.F.Chebotarev Institute of Gerontology of NAMS, Kiev, (886) Department of Internal Diseases-1 of Zaporizhzhya State Medical University, Zaporizhzhya City Clinical Hospital of Emergency Care, Zaporizhzhya, (887) The State Institute of Therapy, L.T. Malaya of Ukrainian National Academy of Medical Science, Kharkov, (888) Policlinic of Administration of Medical Services and Rehabilitation of Artem State Holding, Kiev, (889) State Institution, Ukrainian State Scientific and Research Institute of Medical and Social Problems of Disability of Ministry of Health of Ukraine, Dnipro, (890) National Scientific Center M.D. Strazhesko Institute of Cardiology, Kiev, (891) Communal Institution, Central Clinical Hospital #4 of Zavodsky District, Zaporizhzhia, (892) Kiev City Clinical Hospital #7, Therapeutic dpt #2, Kiev, (893) Medical Center, Desna, Ltd, Ternopil, (894) LTD Cardiology Clinic, Heart and Vessels, Kiev, (895) Clinic of State Institution, Ukrainian State Institute of Medical and Social Problems of Disability Ministry of Public Health, Dnipro, (897) State Institute, Zaporizhzhia Medical Academy of Postgraduate Education of Ministry of Health of Ukraine, Department of Family Medicine With Course of Dermatovenereology and Psychiatry Based On Municipal Institution: Zaporyzhzhya 9th City Multidisciplinary Clinical Hospital, Cardiology Department, Zaporizhzhia, (898) Volyn Regional Clinical Hospital, Department of Cardiosurgery, Lutsk, (899) Zakarpatskyi Oblasnyi Klinichnyi Kardiolohichnyi Dyspanser, m. Uzhhorod, (900) Infarction Dprt of City Clinic Hosp. #6, Simferopol, AR Crimea, (937) Zakarpattya Regional Clinical Cardiology Dispensary, Dept. of General Cardiology, Uzhhorod National University, Chair of Hospital Therapy, Uzhhorod, (938) City Clinical Hospital #1, Vinnitsa, (939) State Institution, National Scientific Center, NAMS Institute of Cardiology M.D. Strazhesko, Department of Essential Hypertension, Kiev, (940) Kharkiv Medical Academy of Postgraduate Education, City Clinical Hospital #8, Kharkiv, (941) Lutsk City Clinical Hospital, Lutsk, (942) Kharkiv City Clinical Hospital #27, Kharkiv, (943) Kharkiv Medical Academy of Postgraduate Education, Kharkiv, (944) Lugansk Regional Cardiological Dispensary, Luhansk, (945) Dnipropetrovsk Medical Academy, Dnipropetrovsk Joint Emergency Hospital, Dnipro, (946) State Institution, National Scientific Center, The M.D. Strazhesko Institute of Cardiology, National Academy of Medical Sciences of Ukraine, Kiev, (947) City Clinical Hospital №3, Chernivtsi, (948) National Institute of Therapy N.A. L. Malaya NAMS, Kharkiv, (949) National Pirogov Memorial Medical University, Vinnytsya, (951) Clinic of State Institution, Institute of Therapy NAMS Ukraine L.T. Maloy, Kharkiv, (952) HSEE of Ukraine, Ukrainian Medical Stomatological Academy, Poltava, (953) Odessa National Medical University, Center of Reconstructive and Recovery Medicine (University Clinic), Odessa, (954) Institute of Urgent and Recovery Surgery, Donetsk, (955) State Institution National Scientific Centre, Acad. M.D. Strazhesko Institute of Cardiology of Nacional Ams of Ukraine, Kiev, (956) Kiev Municipal Clinical Hospital #12, Department of Cardiology; O. O. Bogomolets National Medical University, Kiev, (957) Saint Catherine Odessa, Treatmnent and Diagnostic Center LLC, Odesa, (958) Central City Clinical Hospital #1, Donetsk, (959) Communal Institution, Odesa Regional Cardiological Dispensary, Odesa, (960) Zaporizhzhia Regional Clinical Hospital, Zaporizhzhea, (961) National Scientific Center, NAMS Strazhesko Institute of Cardiology, Kiev, (962) Communal City Clinical Hospital #8, Lviv, (963) Ivano-Frankivsk Regional Clinical

Cardiological Center, Ivano-Frankivsk, (964) City Clinical Hospital #9, Department of Cardiology; State Institution, Dnipropetrovsk Medical Academy of Moh, Dnipro, (965) Kyiv City Clinical Hospital #1, Department of Emergency Cardiology, Kiev, (985) Kyiv City Oleksandrivska Clinical Hospital, Kiev, (992) Cherkasy Regional Cardiological Center, Cherkasy, (994) Kyiv Emergency Care Hospital, Infarction Department, Kiev, (996) The Institute of Gerontology NAMS D.F.Chebotarev, Kiev, (997) D.F. Chebotarev Institute of Gerontology, National Academy of Medical Sciences, Kiev, (998) Odesa Regional Clinical Hospital, Department of Cardiosurgery, Odesa, (999) Ivano-Frankivsk National Medical University, Ivano-Frankivsk

Russian Federation N=709 (850) State Budget Healthcare Institution of City Moscow, City Clinical Hospital N.A. M.P. Konchalovskogo of Healthcare Department, Zelenograd, (851) Saint Petersburg State Budget Healthcare Institution, City Consultative and Diagnostic Center #1, Saint Petersburg, (901) State Health Care Institution City Hospital #117, Saint Petersburg, (902) State Budget Healthcare Institution of Moscow, City Clinical Hospital #4 of The Healthcare Department, Moscow, (903) First Saint Petersburg State Medical University N.A.Acad.I.P.Pavlov of The Ministry of Healthcare of Russian Federation, Saint Petersburg, (904) Pirogov Russian National Research Medical University, Moscow, (905) City Clinical Emergency Hospital#2, Novosibirsk, (906) State Inst City Multidiscipline Hospital# 2, Saint Petersburg, (908) Almazov National Medical Research Centre, Saint Petersburg, (909) Autonomous Non-Profit Organization: Medical Center Alliance, Kirovsk, (910) Central Clinical Hospital of The Russian Academy of Sciences, Moscow, (911) Saint Petersburg State Budget Institution of Healthcare, City Hospital #15, Saint Petersburg, (912) City Clinical Hospital #15 O.M.Filatov, Moscow, (913) FSBHI Clinical Hospital #123 of FMBA, Moscow, (914) Kazan State Medical University, Kazan, (915) Scientific Research Medical Complex, State Budget Institution of Healthcare Clinical Hospital #2, LLC, Kazan, (916) Saint Petersburg State Budget Institution of Healthcare, City Hospital #9, Saint Petersburg, (917) State Budget Healthcare Institution of City Moscow, City Clinical Hospital N.A. V.V.Vinogradova of Healthcare Department, Moscow, (918) International Clinic MEDEM, LLC, Saint Petersburg, (919) Almazov National Medical Research Centre, Saint Petersburg, (921) Saint Petersburg State Budget Institution of Healthcare, City Outpatient Clinic #109, Saint Petersburg, (922) Novosibirsk State Medical University, Novosibirsk, (923) Medinet, LLC, Saint Petersburg, (924) Research Institute of Internal and Preventive Medicine, Branch of The Institute of Cytology and Genetics, Siberian Branch of Russian Academy of Sciences, Novosibirsk, (925) Almazov National Medical Research Centre, Saint Petersburg, (926) Saint Petersburg State Budgetary Healthcare Institution, City Pokrovskaya Hospital, Saint Petersburg, (927) Saratov Regional Veterans Hospital, Saratov, (928) State Healthcare Institution of Yaroslavl Region, Clinical Hospital #8, Yaroslavl, (929) Federal State Budget Military Educational Institution of Higher Professional Education, Military Medical Academy, S.M. Kirov of Ministry of Defence of Russian Federation, Saint Petersburg, (930) The Federal State Autonomous Educational Institution of Higher Education I.M. Sechenov First Moscow State Medical University of Ministry of Healthcare of The Russian Federation (Sechenovskiy University), Moscow, (931) Moscow State Medical and Dental University N.A A.I. Evdokimov of The Ministry of Health, Moscow, (932) FGBU, National Medical Research Center of Cardiology, Ministry of Health

Care of Russia, Moscow, (933) State Budget Institution of Healthcare, Republican Hospital V.A. Baranov, of The Ministry of Healthcare of Karelia Republic, Petrozavodsk, (934) State Budget Healthcare Institution of Arkhangelsk Region, Arkhangelsk Regional Clinical Hospital, Arkhangelsk, (935) Leningrad Regional Cliical Hospital, Saint Petersburg, (936) Science and Research Institute of Experimental Medicine, Saint Petersburg, (966) Saint Petersburg State Budget Healthcare Institution, City Hospital #40 of Kurortniy District, Saint Petersburg, (967) Federal State Budget Institution of Healthcare, Clinical Hospital #122 N.A. L.G. Sokolov Under Federal Medical and Biological Agency of Russia, Saint Petersburg, (968) City Hospital # 38 N A Semashko, Saint Petersburg, (969) State Budget Institution of Healthcare of Arkhangelsk Region, The First City Clinical Hospital E.E. Volosevich, Arkhangelsk, (970) Cardio-Centre, Chernaja Rechka, Saint Petersburg, (971) Central Outpatient Department at Federal State Budget Institution of Healthcare, Northern Medical Clinical Center N.A. Semashko of Federal Medical-Biological Agency, Arkhangelsk, (972) Saint Petersburg State Official Institution of Healthcare, Mariinskaya Ambulatory, Saint Petersburg, (973) Sanatorium Chernaya Rechka, Saint Petersburg, (974) State Healthcare Institution, Regional Clinical Cardiology Dispensary, Saratov, (975) Cardiology Research Institute, Tomsk National Research Medical Center of Russian Academy of Sciences, Tomsk, (976) Research Institute of Complex Issues of Cardiovascular Diseases (NII KPSSZ), Kemerovo, (977) Panaceya Clinic, LLC, Moscow, (978) Nizhny Novgorod Regional Clinical Hospital N.A.Semashko, Nizhny Novgorod, (979) Moscow Hospital #15 O.M.Filatov, Moscow, (980) Budgetary Healthcare Institution of Voronezh Region, Voronezh City Clinical Hospital of Emergency Medical Care #1, Voronezh, (982) Autonomous Healthcare Institution, Voronezh Regional Clinical Consultative and Diagnostic Center, Voronezh, (983) Yaroslavl Regional Clinical Hospital, Yaroslavl, (987) Medical Union New Hospital, LLC, Ekaterinburg, (988) Hospital of Veterans Wars, Kemerovo, (989) State Budget Healthcare Institution of Sverdlovsk Region, Sverdlovsk Regional Clinical Hospital #1, Ekaterinburg, (990) Siberian State Medical University, Tomsk, (991) State Budget Healthcare Institution, Scientific and Research Institute, Regional Clinical Hospital #1 N.A., Krasnodar, (993) Volgograd State Medical University, Department of Therapy and Endocrinology, Volgograd

South Africa N=414 (416) Paarl Research Centre, Paarl, (417) TREAD Research cc, Cape Town, (418) Durbanville Medi-Clinic, Durbanville, (419) Unitas Hospital, Pretoria, (420) Somerset West Clinical Trial Unit, Somerset West, (421) Tiervlei Trial Centre, Bellville, (422) Langeberg Clinical Trials, Cape Town, (423) Synexus Helderberg, Somerset West, (424) Helderberg Research Institute, Somerset West, (425) Vincent Pallotti Hospital, Cape Town, (426) Wits Clinical Research Bara, Soweto, (427) Cardiology Research, Bloemfontein, (430) Newkwa Medical Centre, Durban, (431) Corbod Research Pty Ltd, Panorma, (432) Worthwhile Clinical Trials, Johannesburg, (433) Dr HFM Nortje Clinical Trials, Cape Town, (435) Drs' Joynt Venter and Associates, Witbank, (437) Dr P J Sebastian, Durban, (439) Boanerges Clinical Research, Bloemenstein, Free State, (440) Clinical Projects Research Centre, Worcester, (441) University of Cape Town, Cape Town

Poland N=359 (602) Synexus Polska Sp. Z O.o., Katowice, (603) Synexus Sp. Z O.o., Warsaw, (604) Centrum Medyczne Ogrodowa, Skierniewice, (605) Centrum Medyczne Nzoz As-Medica

Sp.Z O.o., Zgierz, (606) ZSGL LeK, Mikołów, (607) Podlaski Osrodek Kardiologiczny, Bialystok, (608) Institute of Cardiology, Warsaw, (609) Specjalistyczny Gabinet Lekarski Internistyczno-Kardiologiczny Ewa Laskowska-Derlaga, Tarnów, (610) NZOZ SALVIA, Katowice, (612) Centrum Kardiologiczne Pro Corde Sp. Z O.o., Wroclaw, (613) Osteo-Medic Sc, Bialystok, (614) Synexus Sp. Z O.o., Wrocław, (616) Przychodnia Specjalistyczna PROSEN; Department of Experimental and Clinical Physiology, Laboratory of Center for Preclinical Research, Medical University of Warsaw, Warsaw, (617) Centrum Nowoczesnych Terapii, Dobry Lekarz, Krakow

India N=262 (501) G.Kuppuswamy Naidu Memorial Hospital, Coimbatore, Tamilnadu, (502) Medwin Hospitals, Hyderabad, Telangana, (503) JSS Medical College Hospital, Mysuru, Karnataka, (504) Mycure Hospital, Visakhapatnam, Andhra Pradesh, (505) Shatabdi Super Specialty Hospital, Suyojit City Center, Opp. Mahamarg Bus Stand, Nashik, Maharashtra, (506) SR Kalla Memorial Gastro and General Hospital, Jaipur, (507) St. John's Medical College Hospital, Koramangla, Karnataka, (509) KLE Dr. Prabhakar Kore Hospital, Nehru Nagar, (510) Fortis Escorts Heart Institute, New Delhi, (511) Gleneagle Global Hospitals, Hyderabad, Telangana, (513) Monilek Hospital and Research Center, Jaipur, (514) Cardiac Care & Counselling Center, Pune, Maharashtra, (515) Dharma Vira Heart Cente, Sir Ganga Ram Hospital, New Delhi, (516) Vikram Hospital Pvt. Ltd, Mysore, Karnataka, (517) Bhatia Hospital, Mumbai, Maharastra, (518) Endocrinology Diabetes Centre, Bangalore, Karnataka, (524) Caritas Hospital, Kottayam, Kerala, (525) Vikram Hospital, Bangalore, Karnataka, (526) Madras Diabetes Research Foundation, Chennai, Tamilnadu, (527) Krishna Institute of Medical Sciences, Secunderabad, Telanagana

Canada N=250 (172) Recherche Clinique London, Sherbrooke, Québec, (173) Clinique Des Maladies Lipidiques De Québec, Québec, (175) Ronald Collette, MD, Burnaby, (176) DCTM Clinical Trials Group, Strathroy, Ontario, (177) Centre de recherche clinique de Laval, Laval, Québec, (178) SKDS Research Inc., Newmarket, Ontario, (181) Moran Medical Centre, Collingwood, Ontario, (182) Corunna Clinical Research Centre, Corunna, Ontario, (188) London Road Diagnostic Clinic, Sarnia, Ontario, (200) Heathy Heart Program, Vancouver, (201) McGill University Health Centre, Royal Victoria Hospital, Montreal, Québec, (202) Vancouver General Hospital, Vancouver, British Columbia, (219) North Road Clinical Research, Coquitlam, British Columbia, (271) Brampton Research Associates, Brampton, Ontario, (361) Sarnia Institute of Clinical Research, Sarnia, Ontario, (395) Alta Clinical Research Inc, Edmonton, Alberta, (397) Montreal Heart Institute, Montreal, Québec, (659) Centre de Sante et de Services Sociaux de Lanaudière, St-Charles-Borromée, Québec, (660) Institut Universitaire De Cardiologie Et De Pneumologie Du Québec, Université Laval, Québec

Romania N=202 (801) Medical Center Medcalis SRL, Timisoara, (803) Universitary Emergency Hospital, Cardiology Ward I, Bucuresti, (804) Centrul Medical International Bucuresti Dacia, Bucuresti, (805) Emergency County Hospital, Sibiu, (806) Mediclass Sananova SRL, Bucharest, (808) Clinic County Emergency Hospital Cluj-Napoca, Cluj-Napoca, (809) Universitary Emergency Hospital Bucharest, Bucharest, (810) Ilro Medical Clinic, Bucharest, (811) CardioMed SRL, Craiova, (812) Spitalul Judetean De Urgenta Braila, Braila, (813)

University of Medicine and Pharmacy Grigore T. Popa, Rehabilitation Hospital, Department of Cardiovascular Rehabilitation, Lasi, (814) Spitalul Clinic Judetean De Urgenta, Tirgu Mures, (815) Spitalul Clinic Judetean De Urgenta, Sibiu, (816) Centrul Med Plus SRL Bucuresti, Bucuresti, (817) Centrul Medical Galenus, Tirgu Mures, (818) CMI Cardiologie, Baia Mare, (819) SC Clinica Angiomed, Bucharest, (820) Spitalul De Urgenta Arges, Pitesti, (821) CMI Cardiologie, Constanta

Australia N=189 (301) Holy Spirit Northside Hospital, Brisbane, QLD, (302) The Alfred Hospital Heart Centre, Melbourne, VIC, (303) Fremantle Hospital, Fremantle, WA, (304) Dr Heart Pty Ltd, Woolloongabba, QLD, (305) St. Vincents Hospital (Melb) Dept. of Medicine, Fitzroy, VIC, (306) Joondalup Cardiovascular Trials Foundation, Joondalup, WA, (307) Launceston General Hospital, Launceston, TAS, (308) Royal Prince Alfred Hospital, Sydney, NSW, (309) Sir Charles Gairdner Hospital, Nedlands, (310) Royal Perth Hospital, School of Medicine and Pharmacology, Medical Research Foundation, Perth, WA, (311) Lismore Base Hospital Cardiac Catheter Lab, Northern Rivers Cardiovascular Clinic, Lismore, NSW, (312) Barwon Health University Hospital Geelong, Geelong, VIC, (313) Fiona Stanley Hospital, Murdoch, WA, (314) Austin Health, Heidelberg Heights, VIC, (316) Flinders Medical Centre and Flinders University, Bedford Park, SA, (317) Cardiovascular Trials WA (South), Bateman, WA, (319) Core Research Group Pty Ltd, Brisbane, QLD

New Zealand N=134 (402) Lipid and Diabetes Research, Christchurch, (405) Waitemata District Health Board, Diabetes Service, Auckland, (406) Cardiology Department, Nelson Hospital, Nelson, (408) Hawke's Bay District Health Board, Hastings, (411) Waitemata District Health Board, Auckland, (414) Cardiology Clinical Trials Unit, Waikato District Health Board, Hamilton, (415) Southern District Health Board, Dunedin Hospital, Dunedin, (442) Taranaki Base Hospital, New Plymouth, (444) Northland District Health Board, Whangarei, (445) Bay of Plenty Clinical Trials Unit, Tauranga

Figure S1. Efficacy and Safety of Icosapent Ethyl Among Patients with Prior PCI, Stratified by Baseline Antithrombotic Regimen.

Endpoint/Subgroup	Icosapent Ethyl	Placebo	Icosapent Ethyl vs. Placebo	P-value	Int. P-value
	n/N (%)	*n/N (%)*	*HR (95% CI)*		
**Primary Composite Endpoint**	362/1737 (20.8)	491/1671 (29.4)	0.66 (0.58, 0.76)	<0.0001	
**Baseline Medications**					0.33
Aspirin Only	150/873 (17.2)	217/796 (27.3)	0.59 (0.48, 0.73)	<0.0001	
Aspirin AND (Clopidogrel OR Prasugrel OR Ticagrelor)	146/618 (23.6)	185/612 (30.2)	0.74 (0.60, 0.92)	0.007	
Warfarin OR Rivaroxaban OR Apixaban	13/31 (41.9)	9/28 (32.1)	1.02 (0.43, 2.42)	0.97	
Both Anticoagulants + Any Anti-Platelet Above	16/49 (32.7)	23/52 (44.2)	0.72 (0.38, 1.38)	0.32	
**Key Secondary Composite Endpoint**	208/1737 (12.0)	290/1671 (17.4)	0.66 (0.56, 0.79)	<0.0001	
**Baseline Medications**					0.52
Aspirin Only	91/873 (10.4)	135/796 (17.0)	0.60 (0.46, 0.78)	0.0001	
Aspirin AND (Clopidogrel OR Prasugrel OR Ticagrelor)	74/618 (12.0)	100/612 (16.3)	0.71 (0.53, 0.96)	0.03	
Warfarin OR Rivaroxaban OR Apixaban	11/31 (35.5)	7/28 (25.0)	1.07 (0.41, 2.84)	0.89	
Both Anticoagulants + Any Anti-Platelet Above	10/49 (20.4)	15/52 (28.8)	0.78 (0.35, 1.74)	0.54	
**Any Bleeding**	221/1737 (12.7)	202/1671 (12.1)	1.03 (0.85, 1.25)	0.73	
**Baseline Medications**					0.008
Aspirin Only	81/873 (9.3)	93/796 (11.7)	0.77 (0.57, 1.04)	0.08	
Aspirin AND (Clopidogrel OR Prasugrel OR Ticagrelor)	90/618 (14.6)	70/612 (11.4)	1.30 (0.95, 1.78)	0.09	
Warfarin OR Rivaroxaban OR Apixaban	5/31 (16.1)	7/28 (25.0)	0.33 (0.10, 1.09)	0.06	
Both Anticoagulants + Any Anti-Platelet Above	15/49 (30.6)	7/52 (13.5)	2.35 (0.95, 5.78)	0.06	
**Serious Bleeding**	56/1737 (3.2)	46/1671 (2.8)	1.15 (0.78, 1.69)	0.49	
**Baseline Medications**					0.38
Aspirin Only	17/873 (1.9)	16/796 (2.0)	0.94 (0.47, 1.85)	0.85	
Aspirin AND (Clopidogrel OR Prasugrel OR Ticagrelor)	20/618 (3.2)	15/612 (2.5)	1.38 (0.70, 2.70)	0.35	
Warfarin OR Rivaroxaban OR Apixaban	2/31 (6.5)	3/28 (10.7)	0.51 (0.08, 3.21)	0.47	
Both Anticoagulants + Any Anti-Platelet Above	7/49 (14.3)	3/52 (5.8)	2.52 (0.65, 9.76)	0.17	

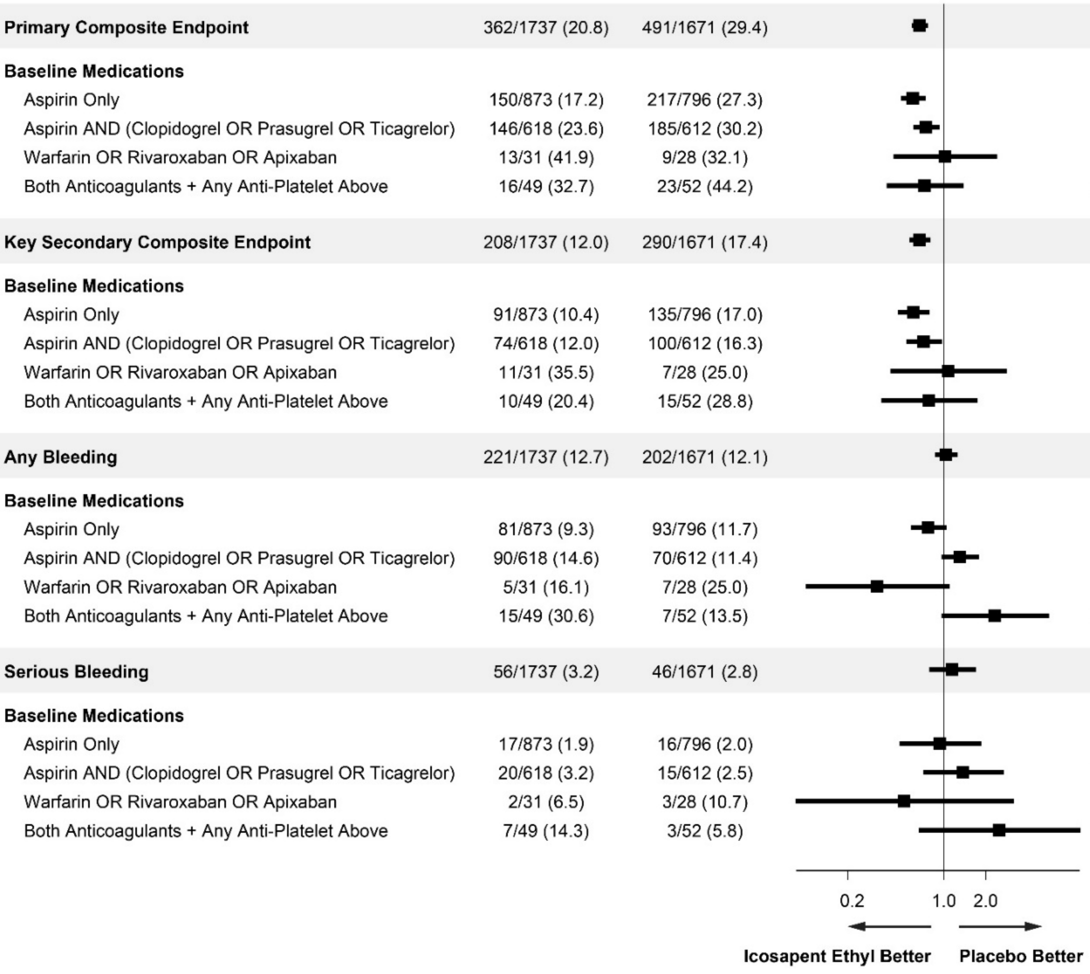

Figure S2. Forest Plot of Efficacy End Points in Hierarchical Testing Order by Sex Among Patients with Prior PCI.

Endpoint/Subgroup	Icosapent Ethyl	Placebo	Icosapent Ethyl vs. Placebo	P-value	Int. P-value
	n/N (%)	*n/N (%)*	*HR (95% CI)*		
**Primary Composite Endpoint**	362/1737 (20.8)	491/1671 (29.4)	0.66 (0.58, 0.76)	<0.0001	
**Gender**					0.88
Male	298/1387 (21.5)	398/1317 (30.2)	0.66 (0.57, 0.77)	<0.0001	
Female	64/350 (18.3)	93/354 (26.3)	0.70 (0.51, 0.97)	0.03	
**Key Secondary Composite Endpoint**	208/1737 (12.0)	290/1671 (17.4)	0.66 (0.56, 0.79)	<0.0001	
**Gender**					0.81
Male	172/1387 (12.4)	233/1317 (17.7)	0.67 (0.55, 0.82)	<0.0001	
Female	36/350 (10.3)	57/354 (16.1)	0.66 (0.43, 1.01)	0.05	
**Cardiovascular Death or Nonfatal Myocardial Infarction**	181/1737 (10.4)	248/1671 (14.8)	0.68 (0.56, 0.82)	<0.0001	
**Gender**					0.69
Male	151/1387 (10.9)	200/1317 (15.2)	0.69 (0.56, 0.86)	0.0006	
Female	30/350 (8.6)	48/354 (13.6)	0.66 (0.41, 1.04)	0.07	
**Fatal or Nonfatal Myocardial Infarction**	141/1737 (8.1)	198/1671 (11.8)	0.66 (0.53, 0.82)	0.0002	
**Gender**					0.81
Male	120/1387 (8.7)	168/1317 (12.8)	0.65 (0.52, 0.83)	0.0003	
Female	21/350 (6.0)	30/354 (8.5)	0.72 (0.41, 1.26)	0.25	
**Urgent or Emergent Revascularization**	144/1737 (8.3)	205/1671 (12.3)	0.65 (0.52, 0.80)	<0.0001	
**Gender**					1.00
Male	123/1387 (8.9)	172/1317 (13.1)	0.64 (0.51, 0.81)	0.0002	
Female	21/350 (6.0)	33/354 (9.3)	0.64 (0.37, 1.12)	0.11	
**Cardiovascular Death**	55/1737 (3.2)	81/1671 (4.8)	0.64 (0.46, 0.90)	0.01	
**Gender**					0.91
Male	41/1387 (3.0)	59/1317 (4.5)	0.66 (0.44, 0.98)	0.04	
Female	14/350 (4.0)	22/354 (6.2)	0.68 (0.34, 1.34)	0.26	
**Hospitalization for Unstable Angina**	75/1737 (4.3)	118/1671 (7.1)	0.59 (0.44, 0.79)	0.0003	
**Gender**					0.66
Male	62/1387 (4.5)	92/1317 (7.0)	0.60 (0.44, 0.83)	0.002	
Female	13/350 (3.7)	26/354 (7.3)	0.51 (0.26, 1.01)	0.05	
**Fatal or Nonfatal Stroke**	39/1737 (2.2)	59/1671 (3.5)	0.62 (0.41, 0.92)	0.02	
**Gender**					0.46
Male	28/1387 (2.0)	45/1317 (3.4)	0.57 (0.35, 0.91)	0.02	
Female	11/350 (3.1)	14/354 (4.0)	0.83 (0.38, 1.84)	0.65	
**Total Mortality/Nonfatal Myocardial Infarction/Nonfatal Stroke**	255/1737 (14.7)	325/1671 (19.4)	0.72 (0.61, 0.85)	0.0001	
**Gender**					0.78
Male	204/1387 (14.7)	258/1317 (19.6)	0.72 (0.60, 0.86)	0.0004	
Female	51/350 (14.6)	67/354 (18.9)	0.78 (0.54, 1.12)	0.17	
**Total Mortality**	108/1737 (6.2)	124/1671 (7.4)	0.82 (0.63, 1.06)	0.13	
**Gender**					0.70
Male	78/1387 (5.6)	91/1317 (6.9)	0.80 (0.59, 1.08)	0.15	
Female	30/350 (8.6)	33/354 (9.3)	0.92 (0.56, 1.52)	0.74	

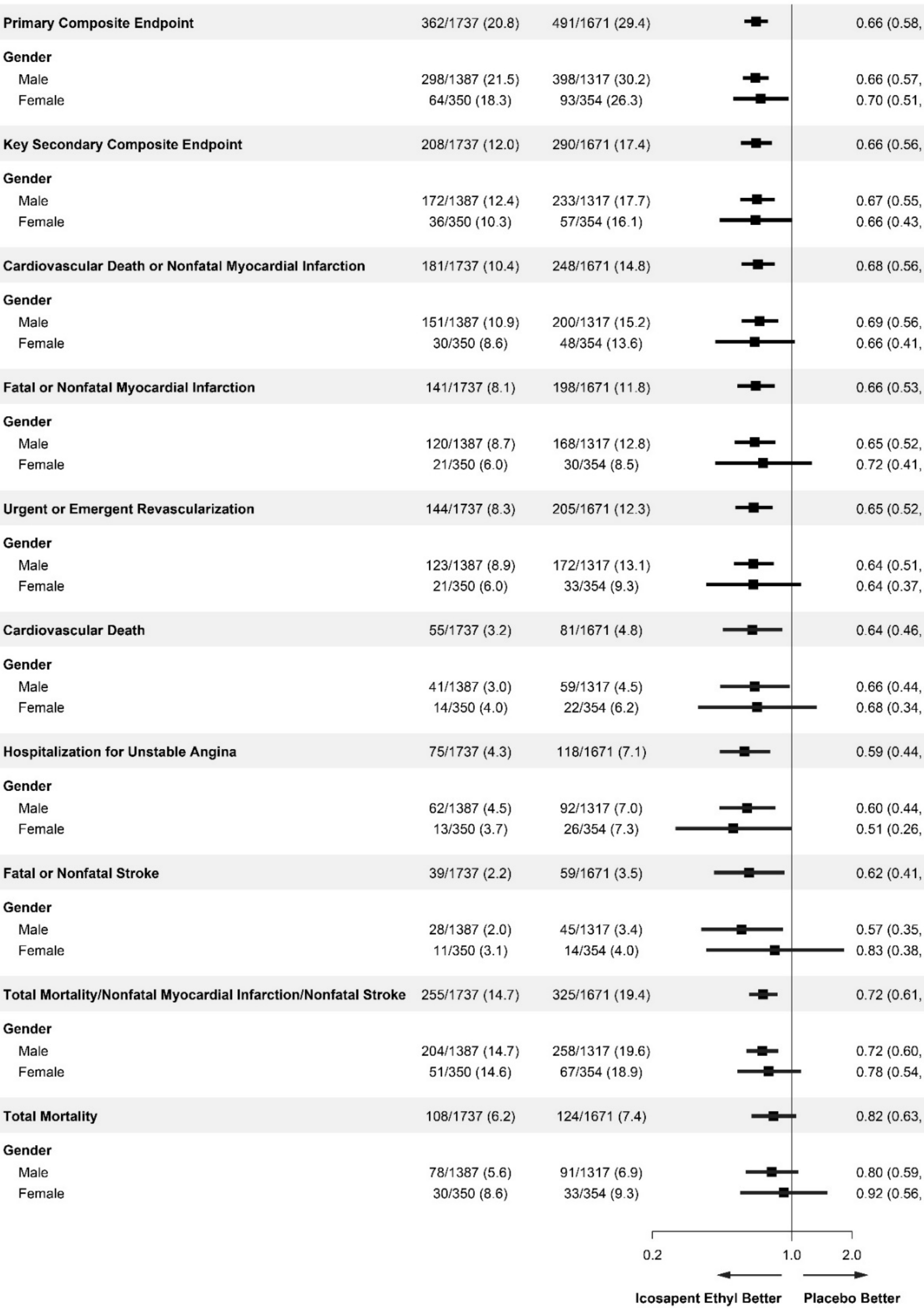

Figure S3. Cumulative Incidence Plots of A. Primary Composite End Point and B. Key Secondary Composite End Point with Non-CV Death as Competing Risk Among Patients with Prior PCI.

A.

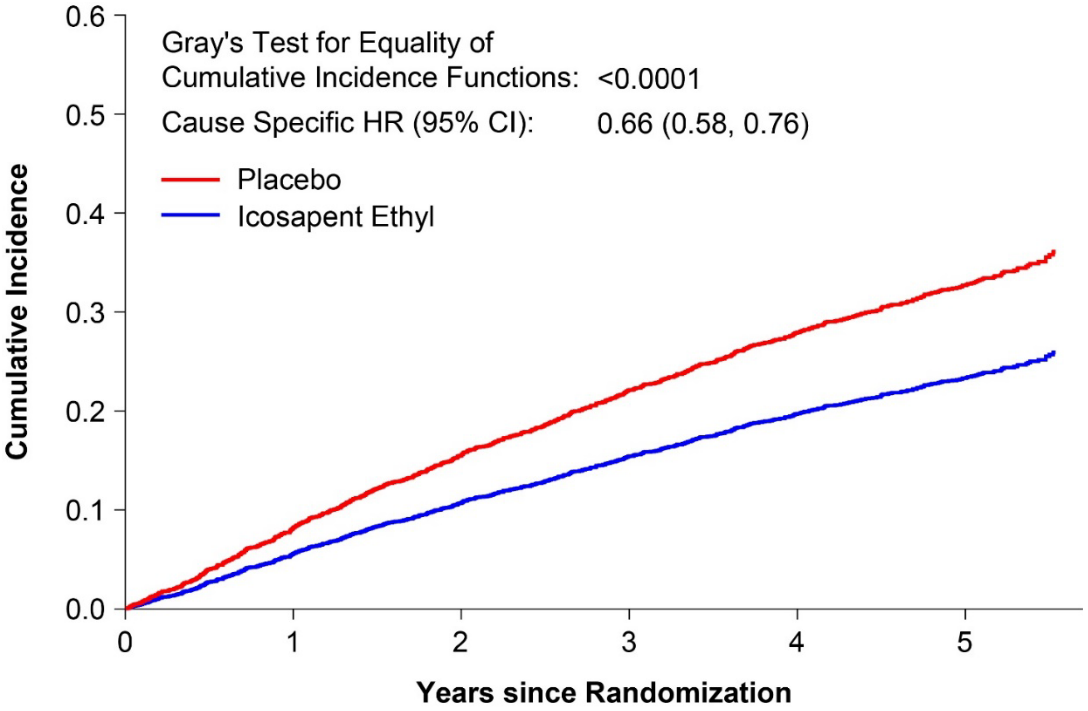

B.

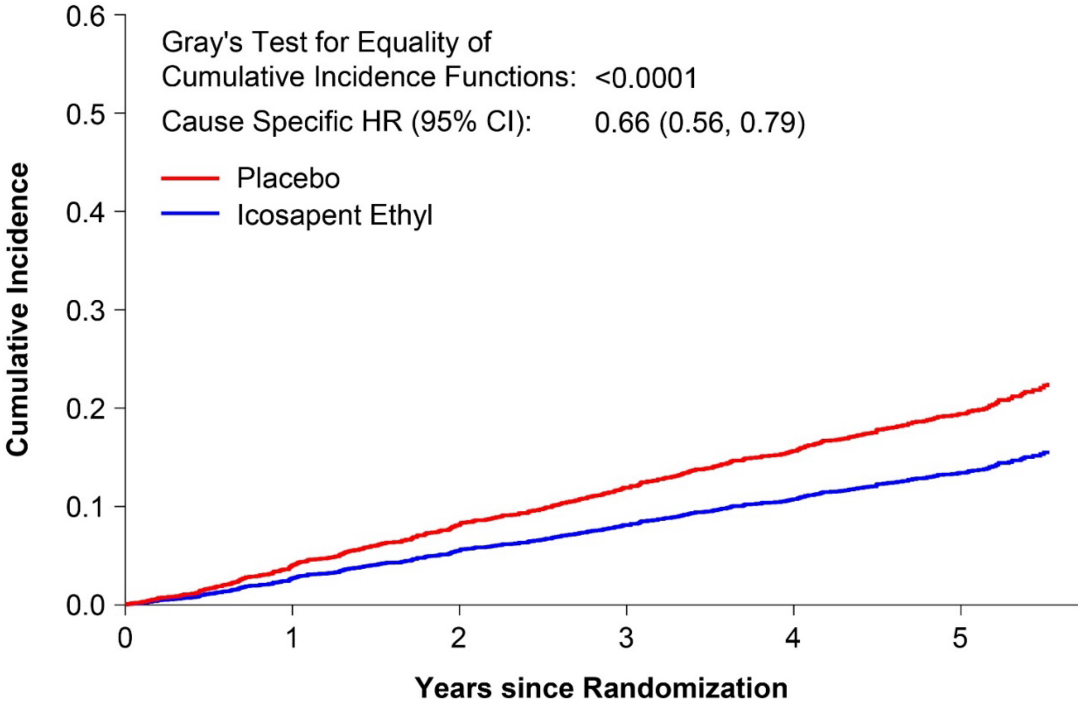

Figure S4. Forest Plot of Primary and Key Secondary Composite End Points for Patients With or Without Prior PCI

Endpoint/Subgroup	Icosapent Ethyl	Placebo	Icosapent Ethyl vs. Placebo	P-value	Interaction P-value
	n/N (%)	*n/N (%)*	*HR (95% CI)*		
**Primary Composite Endpoint**					0.11
Prior PCI	362/1737 (20.8)	491/1671 (29.4)	0.66 (0.58, 0.76)	<0.0001	
ASCVD w/o PCI	215/1248 (17.2)	281/1310 (21.5)	0.79 (0.66, 0.95)	0.01	
**Key Secondary Composite Endpoint**					0.39
Prior PCI	208/1737 (12.0)	290/1671 (17.4)	0.66 (0.56, 0.79)	<0.0001	
ASCVD w/o PCI	159/1248 (12.7)	220/1310 (16.8)	0.75 (0.61, 0.91)	0.005	

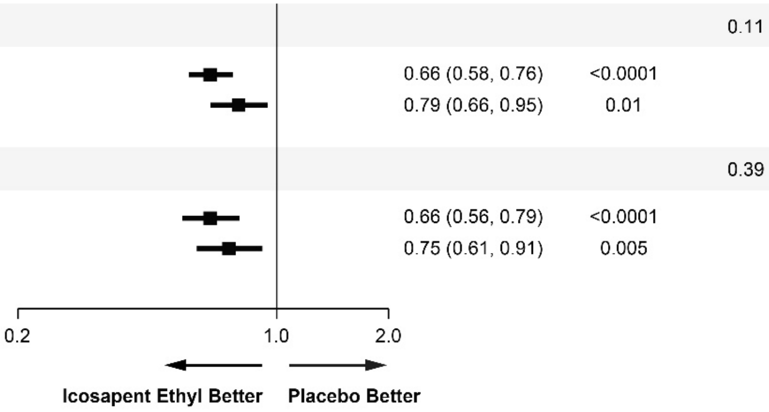

Supplement: Supplementary file 1 — Appendix S1. REDUCE‐IT Investigators Figures S1–S4 [file JAH3-11-e022937-s001.pdf]
